# β-Nitrostyrenes as Potential Anti-leishmanial Agents

**DOI:** 10.3389/fmicb.2016.01379

**Published:** 2016-09-01

**Authors:** Syed Shafi, Farhat Afrin, Mohammad Islamuddin, Garima Chouhan, Intzar Ali, Faatima Naaz, Kalicharan Sharma, Mohammad S. Zaman

**Affiliations:** ^1^Medicinal Chemistry Lab, Department of Chemistry, Faculty of Science, Hamdard UniversityNew Delhi, India; ^2^Department of Medical Laboratories Technology, Faculty of Applied Medical Sciences, Taibah UniversityMedina, Saudi Arabia; ^3^Parasite Immunology Lab, Department of Biotechnology, Faculty of Science, Hamdard UniversityNew Delhi, India; ^4^Molecular Virology and Vaccinology Lab, Department of Biotechnology, Faculty of Science, Hamdard UniversityNew Delhi, India; ^5^Membrane Biology Laboratory, School of Life Sciences, Jawaharlal Nehru UniversityNew Delhi, India; ^6^Medicinal Chemistry Lab, Department of Pharmaceutical Chemistry, Faculty of Pharmacy, Jamia HamdardNew Delhi, India

**Keywords:** β-nitrostyrenes, leishmanicidal, promastigotes, antimicrobicidal, macrophages

## Abstract

Development of new therapeutic approach to treat leishmaniasis has become a priority. In the present study, the antileishmanial effect of β-nitrostyrenes was investigated against *in vitro* promastigotes and amastigotes. A series of β-nitrostyrenes have been synthesized by using Henry reaction and were evaluated for their antimicrobial activities by broth microdilution assay and *in vitro* antileishmanial activities against *Leishmania donovani* promastigotes by following standard guidelines. The most active compounds were futher evaluated for their *in vitro* antileishmanial activities against intracellular amastigotes. Among the tested β-nitrostyrenes, compounds 7, 8, 9, 12, and 17 exhibited potential activities (MICs range, 0.25–8 μg/mL) against clinically significant human pathogenic fungi. However, the microbactericidal concentrations (MBCs) and the microfungicidal concentrations (MFCs) were found to be either similar or only two-fold greater than the MICs. Anti-leishmanial results demonstrated that compounds 9, 12, 14, and 18 were found to be most active among the tested samples and exhibited 50% inhibitory concentration (IC_50_) by 23.40 ± 0.71, 37.83 ± 3.74, 40.50 ± 1.47, 55.66 ± 2.84 nM against *L. donovani* promastigotes and 30.5 ± 3.42, 21.46 ± 0.96, 26.43 ± 2.71, and 61.63 ± 8.02 nM respectively against intracellular *L. donovani* promastigotes amastigotes respectively which are comparable with standard AmB (19.60 ± 1.71 nM against promastigotes and 27.83 ± 3.26 nM against amastigotes). Compounds 9, 12, 14, and 18 were found to have potent *in vitro* leishmanicidal activity against *L. donovani* and found to be non-toxic against mammalian macrophages even at a concentration of 25 μM. Nitric oxide (NO) estimation studies reveals that these compounds are moderately inducing NO levels.

## Introduction

Leishmaniasis is a complex infection caused by multiple species of the intracellular protozoan parasites of genus *Leishmania* and is transmitted by the bite of a female phlebotomine sand fly vector. The disease is endemic in 82 countries of tropical and subtropical areas around the world and affects an estimated 12 million people with 2 million new cases annually (Davies et al., 2003; Murray et al., 2005; WHO, 2005, 2007a; Piscopo and Azzopardi, 2007). It is one of the most neglected tropical diseases, with a major impact among the poorest. Visceral leishmaniasis (VL) is also known as kala-azar (black fever) in the Indian sub-continent which is the most severe form of the disease, is being fatal if untreated (WHO, 1999; Gour et al., 2009). In non-endemic areas of the world, VL is mostly an opportunistic infection and often associated with human immunodeficiency virus (HIV) infection (WHO, 2007b, 2010).

The number of leishmaniasis cases is increasing worldwide due to the lack of safe and effective drug, vaccines, difficulty in controlling vectors and mounting parasite resistance. From the 1940s, pentavalent antimonials (Sodium antimony gluconate-SAG, sodium stibogluconate-pentostam, and meglumine antimoniate-glucantime) are being used to prevent leishmaniasis since their discovery (Croft et al., 2005; Mishra et al., 2007; Santos et al., 2008). These drugs are highly toxic and require prolonged treatments with parenteral administration (Croft et al., 2005; WHO, 2010). Further the escalating antimonials resistance to strains of *Leishmania donovani* in India, makes most of the cases un-responsive to treatment (Croft et al., 2005; Gour et al., 2009). The inefficacy of antimonials is particularly prominent in HIV-Leishmania co-infections (Croft et al., 2005; Mishra et al., 2007). Over the past decades, few alternative drugs including pentamidine, isethionate, amphotericin B, paromomycin, miltefosine have been developed. But none of them are ideal for treatment due to high toxicity, resistance, exorbitant prices, prolonged treatment and inadequate mode of administration (Davies et al., 2003; Kayser et al., 2003; Croft et al., 2005; Alvar et al., 2006; Mishra et al., 2007; Santos et al., 2008; Sundar et al., 2011). The adverse effects and unaffordable prices of the current drugs divulge an urgent need for new, safer, effective, and cheaper alternates (Kayser et al., 2003).

Evaluation of antimicrobial agents against leishmanial parasites is being one of the promising approaches in the leishmanial drug discovery. Antifungal agents have shown some positive results in this prospective (Verma et al., 2011; Ibrahim et al., 2012). Azole antifungal agents have been used as antileishmanial agents since 1980s, (Saenz et al., 1990; Alrajhi et al., 2002; Kashani et al., 2005; Murray et al., 2005) but their use in the treatment of cutaneous and VL has produced inconsistent results (Weinrauch et al., 1987; Beach et al., 1988; Hart et al., 1989; Wali et al., 1990; Urbina, 1997; Al-Abdely et al., 1999). Further in 1981, New et al. investigated parasiticidal activity of a number of anti-fungal agents including: griseofulvin, 5-fluorocytosine, and amphotericin B against VL (New et al., 1981). Amphotericin B is emerged as a standalone drug which is the only drug in clinical use for VL. But the adverse effects and increasing resistance of Amphotericin B stating the need for the development of new chemical entities. In this regard we focused at the development of natural product based new anti-leishmanial agents from the known anti-fungal agents.

β-nitrostyrenes have been known for their antimicrobial activities over the past few decades (Brian et al., 1946; Schales and Graefe, 1952; Evans et al., 1956; Huitric et al., 1956; Worthen and Bond, 1970; Mikami et al., 1987, 1991; Denisenko et al., 2004; Milhazes et al., 2006; Al-Zereini et al., 2007; Pettit et al., 2009; Lo et al., 2012). Their antifungal properties have also been known over the years and demonstrated a broad spectrum of activities against various fungal strains (Schales and Graefe, 1952; Evans et al., 1956; Huitric et al., 1956; Mikami et al., 1987, 1991; Al-Zereini et al., 2007; Pettit et al., 2009).

**Figure 1:**
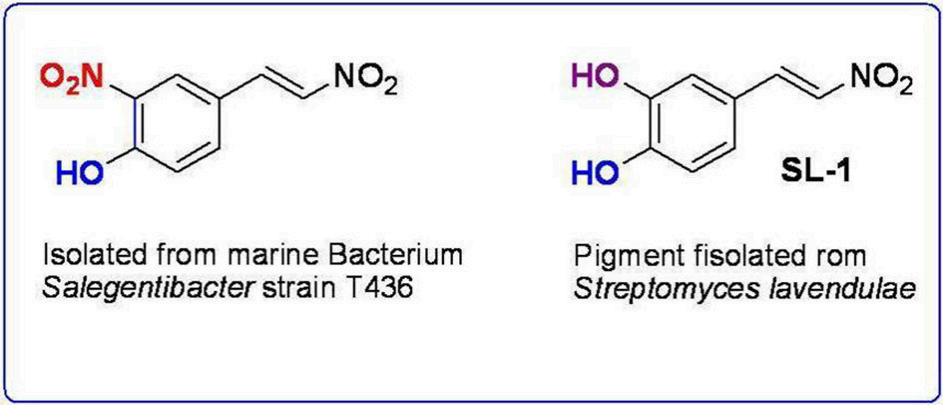
**Naturally occurring β-nitrostyrenes**.

β-nitrostyrenes have been rarely found in nature. 3,4-dihydroxy-β-nitrostyrene (SL-1), a reddish brown compound was isolated from *Streptomyces lavendulaea* (Mikami et al., 1987). 2-nitro-4-((*E*)-2-nitrovinyl)phenol, an another β-nitrostyrene has been isolated from an Indian mangrove plant *Sonneratia acids* Linn. F (Mikami et al., 1991). The same β-nitrostyrene has been further isolated from *Salegentibacter* sp. T436 an Artic sea ice bacterium (Al-Zereini et al., 2007). Both the natural β-nitrostyrenes (Figure 1) and their synthetic derivatives were found to exhibit a wide range of antibacterial and antifungal activities (Brian et al., 1946; Schales and Graefe, 1952; Evans et al., 1956; Huitric et al., 1956; Worthen and Bond, 1970; Mikami et al., 1987, 1991; Denisenko et al., 2004; Milhazes et al., 2006; Al-Zereini et al., 2007; Pettit et al., 2009; Lo et al., 2012).

Apart from the antimicrobial properties, some β-nitrostyrene derivatives found to be anti-proliferative agents (Carter et al., 2002; Kaap et al., 2003), selective human telomerase inhibitors (Kim et al., 2003), antiplatelet (Hsieh et al., 2010) agents and NLRP3 activation inhibitors (He et al., 2014). The nitrovinyl side chain attached to the aromatic ring was recognized to be an essential chemical feature in this type of compounds and a critical conformational pattern for their biological activity (Evans et al., 1956; Huitric et al., 1956; Mikami et al., 1987, 1991; Milhazes et al., 2006; Al-Zereini et al., 2007).

The promising antifungal properties of β-nitrostyrenes and the morphological similarities of fungi with leishmanial parasite prompted us to evaluate the anti-leishmanial potential of β-nitrostyrenes and to develop a structure activity relationship (SAR) around this nitrovinyl moiety. In the present report we are presenting the anti-leishmanial potential of β-nitrostyrenes for the first time.

## Materials and methods

### Animals

BALB/c mice was used for the isolation of peritoneal macrophages in the *ex vivo* anti-amastigote study after prior approval from the Jamia Hamdard Animal Ethics Committee (JHAEC). JHAEC is registered under the Committee for the purpose of supervision and control of experiments on animals (CPCSEA). Mice were individually housed in the Central Animal House of Jamia Hamdard as per internationally accepted norms. The mice were kept in standard size polycarbonate cages under controlled conditions of temperature (23 ± 1°C), humidity (55 ± 10%), 12:12 h of light and dark cycle and fed with standard pellet diet and filtered water (*ad libitum*).

### Materials

All commercial chemicals used as starting materials and reagents in this study were purchased from Merck (India), Spectrochem, and Sigma Aldrich which were of reagent grade. All melting points were uncorrected and measured using Electro-thermal IA 9100 apparatus (Shimadzu, Japan); IR spectra were recorded on Bruker ALPHA FT-IR spectrometer (Germany), ^1^H- NMR spectra were determined on a Bruker (300 and 400 MHz) spectrometer and chemical shifts were expressed as ppm against TMS as internal reference. Mass spectra were recorded on 70 eV (EI Ms-QP 1000EX, Shimadzu, Japan), Column Chromatography was performed on (Merck) Silica gel 60 (particle size 0.06e 0.20 mm).

#### General procedure for the synthesis of β-nitrostyrenes

All the nitrostyrenes were synthesized using the known Henry reaction. To the stirred solution of acetic acid (5 mL) and ammonium acetate (20 eq to aldehyde) was added nitromethane/nitroethane/nitropropane (2 eq to aldehyde) followed by aromatic aldehyde (1 eq) and the solution was refluxed in an oil bath at 110°C for 6-8 h. After the clearance of TLC, the total dark solution was distilled under *vacume* completely brown colored syrup obtained, the syrup was poured in crushed ice and product was extracted with ethyl acetate (50 mL × 3), the combined organic layer dried over Na_2_SO_4_. The combined organic layers were evaporated under vacuum to afford the impure reaction mixture which was purified by column chromatography (silica gel) to obtain the pure β-nitrostyrene.

1. *1-chloro-4-((E)-2-nitrovinyl) benzene (1)* (Kumar et al., 2012): IR (cm^−1^): 3092, 2959, 2348, 1624, 1484, 1398, 1330, 1251, 1077, 965, 812, 735; ^1^H NMR (DMSO-D6,300 MHz): δ 7.53 (d, *J* = 8.4 Hz, 2H), 7.86 (d, *J* = 8.7 Hz, 2H), 8.11 (d, *J* = 13.8 Hz, 1H), 8.23 (d, *J* = 13.5 Hz, 1H); ESI-MS (m/z): 184 (M^+^+1); Anal. Calcd for (C_8_H_6_ClNO_2_): C, 52.34; H, 3.29; Cl, 19.31; N, 7.63. Found: C, 52.35; H, 3.32; Cl, 19.27; N, 7.61.
2. *1-chloro-4-((E)-2-nitroprop-1-enyl) benzene (2)* (Cornell et al., 2014): IR (cm^−1^): 3180, 2980, 1653, 1503, 1397, 1299, 1081, 973, 816; ^1^H NMR (DMSO-D6,300 MHz): δ 2.37 (s, 3H), 7.30 (d, *J* = 8.4 Hz, 2H), 7.37 (d, *J* = 8.7 Hz, 2H), 7.97 (s, 1H); ESI-MS (m/z): 198 (M^+^+1); Anal. Calcd for (C_9_H_8_ClNO_2_): C, 54.70; H, 4.08; Cl, 17.94; N, 7.09. Found: C, 54.67; H, 4.11; Cl, 17.92; N, 7.08.
3. *1-chloro-4-((E)-2-nitrobut-1-enyl) benzene (3)*: IR (cm^−1^): 2972, 2935, 1643, 1589, 1549, 1517, 1486, 1302, 1083, 1005, 918, 823, 794; ^1^H NMR (CDCl_3_,400 MHz): δ 1.27 (t, *J* 7.6 Hz, 3H), 2.84 (q, *J* = 7.6 Hz, 2H), 7.35 (d, *J* = 8.4 Hz, 2H), 7.43 (d, *J* = 8.4 Hz, 2H), 7.95 (s, 1H); ^13^C NMR (CDCl_3_,100 MHz): 12.34, 21.05, 112.48, 118.60, 133.15, 135.15, 147.55, 152.21; ESI-MS (m/z): 212 (M^+^+2), 213 (M^+^+2); Anal. Calcd for (C_10_H_10_ClNO_2_): C, 56.75; H, 4.76; Cl, 16.75; N, 6.62. Found: C, 56.78; H, 4.73; Cl, 16.72; N, 6.59.
4. *N,N-dimethyl-4-((E)-2-nitrovinyl) benzenamine (4)* (Wang and Wang, 2002): IR (cm^−1^): 2939, 1574, 1474, 1230, 1061, 963, 796; ^1^H NMR (DMSO-D_6_, 300 MHz): δ 3.03 (s, 6H), 6.74 (d, *J* = 8.7 Hz, 2H), 7.66 (d, *J* = 8.7 Hz, 2H), 7.96 (d, *J* = 13.2 Hz, 1H), 8.02 (d, *J* = 13.2 Hz, 1H); ESI-MS (m/z): 193 (M^+^+1); Anal. Calcd for (C_10_H_12_N_2_O_2_): C, 62.49; H, 6.29; N, 14.57. Found: C, 62.52; H, 6.28; N, 14.55.
5. *N,N-dimethyl-4-((E)-2-nitroprop-1-enyl) benzenamine (5)* (Yan et al., 2008): IR (cm^−1^): 2896, 2358, 1592, 1480, 1359, 1228, 1164, 859, 809; ^1^H NMR (CDCl_3_,400 MHz): δ 2.49 (s, 3H), 3.05 (s, 6H), 6.72 (d, *J* = 9.2 Hz, 2H), 7.40 (d, *J* = 8.0 Hz, 2H), 8.08 (s, 1H); ESI-MS (m/z): 207 (M^+^+1); Anal. Calcd for (C_11_H_14_N_2_O_2_): C, 64.06; H, 6.84; N, 13.58. Found: C, 64.02; H, 6.87; N, 13.59.
6. *N,N-dimethyl-4-((E)-2-nitrobut-1-enyl) benzenamine (6)*: IR (cm^−1^): 2972, 2364, 1605, 1526, 1056; ^1^H NMR (CDCl_3_,400 MHz): δ 1.28 (t, *J* = 7.2 Hz, 3H), 2.93 (q, *J* = 7.2 Hz, 2H), 3.05 (s, 6H), 6.72 (d, *J* = 9.2 Hz, 2H), 7.39 (d, *J* = 8.8 Hz, 2H), 8.03 (s, 1H); ESI-MS (m/z): 220 (M^+^), 221 (M^+^+1); Anal. Calcd for (C_12_H_16_N_2_O_2_): C, 65.43; H, 7.32; N, 12.72. Found: C, 65.46; H, 7.33; N, 12.68.
7. *1-nitro-4-((E)-2-nitrovinyl) benzene (7)* (Kumar et al., 2012): IR (cm^−1^): 3110, 2966, 2879, 2358, 1697, 1517; ^1^H NMR (CDCl_3_,400 MHz): δ 7.64-7.70 (m, 2H), 7.87 (d, *J* = 7.6 Hz, 1H), 8.05 (d, *J* = 13.6 Hz, 1H), 8.35 (d, *J* = 8.4 Hz, 2H); ESI-MS (m/z): 195 (M^+^+1); Anal. Calcd for (C_8_H_6_N_2_O_4_): C, 49.49; H, 3.12; N, 14.43. Found: C, 49.46; H, 3.09; N, 14.46.
8. *1-nitro-4-((E)-2-nitroprop-1-enyl) benzene (8)*: IR (cm^−1^): 2925, 2360, 1692, 1521, 1342, 800; ^1^H NMR (CDCl_3_,400 MHz): δ 2.45 (s, 3H), 7.58 (d, *J* = 8.00 Hz, 2H), 8.08 (s, 1H), 8.31 (d, *J* = 8.70 Hz, 2H), ESI-MS (m/z): 208 (M^+^), 209 (M^+^+1); Anal. Calcd for (C_9_H_8_N_2_O_4_): C, 51.93; H, 3.87; N, 13.46. Found: C, 51.94; H, 3.91; N, 13.43.
9. *1-nitro-4-((E)-2-nitrobut-1-enyl) benzene (9)*: IR (cm^−1^): 2925, 1692, 1521, 1342, 800; ^1^H NMR (DMSO-D6,300 MHz): δ 1.18 (t, *J* = 7.20 Hz, 3H), 2.78 (q, *J* = 7.20 Hz), 7.81 (d, *J* = 8.70 Hz, 2H), 8.14 (s, 1H), 8.32 (d, *J* = 8.7 Hz, 2H); ESI-MS (m/z): 222 (M^+^), 223 (M^+^+1); Anal. Calcd for (C_10_H_10_N_2_O_4_): C, 54.05; H, 4.54; N, 12.61. Found: C, 54.08; H, 4.51; N, 12.59.
10. *1-nitro-3-((E)-2-nitrovinyl)benzene (10)* (Manna et al., 2013): IR (cm^−1^): 3003, 2875, 1694, 1521, 1057, 854; ^1^H NMR (CDCl_3_, 400 MHz): δ 7.51 (t, *J* = 8.0 Hz, 1H), 7.69-7.71 (m, 1H), 7.72 (d, *J* = 12.0 Hz, 1H), 8.07–8.10 (m, 1H), 8.18 (d, *J* = 12.0 Hz), 8.28 (s, 1H); ESI-MS (m/z): 195 (M^+^+1); Anal. Calcd for (C_8_H_6_N_2_O_4_): C, 49.49; H, 3.12; N, 14.43. Found: C, 49.53; H, 3.07; N, 14.48.
11. *1-nitro-3-((E)-2-nitrobut-1-enyl) benzene (11)* (Yan et al., 2008): IR (cm^−1^): 3088, 2875, 1664, 1526, 1344, 903, 811, 718; ^1^H NMR (CDCl_3_, 400 MHz): δ 2.69 (s, 3H), 7.67 (t, *J* = 8.0 Hz, 1H), 7.98 (d, *J* = 7.6 Hz, 1H), 8.19 (s, 1H), 8.35 (d, *J* = 8.00 Hz, 1H), 8.39 (s, 1H); ESI-MS (m/z): 209 (M^+^+1); Anal. Calcd for (C_9_H_8_N_2_O_4_): C, 51.93; H, 3.87; N, 13.46. Found: C, 51.89; H, 3.97; N, 13.48.
12. *1-nitro-3-((E)-2-nitrobut-1-enyl) benzene (12)*: IR (cm^−1^): 2925, 2362, 2327, 1696, 1524, 1344; ^1^H NMR (DMSO-D6,300 MHz): δ 1.20 (t, *J* = 7.20 Hz, 3H), 2.80 (q, *J* = 7.2 Hz, 2H), 7.80 (t, *J* = 8.1 Hz, 1H), 7.98 (d, *J* = 7.5 Hz, 1H), 8.17 (s, 1H), 8.31 (d, *J* = 8.10 Hz, 1H), 8.37 (s, 1H); ^13^C NMR (CDCl_3_,75 MHz): 12.45, 20.74, 124.03, 124.37, 130.19, 130.26, 134.07, 135.17, 148.50, 155.36; ESI-MS (m/z): 222 (M^+^), 240 (M^+^+18); Anal. Calcd for (C_10_H_10_N_2_O_4_): C, 54.05; H, 4.54; N, 12.61. Found: C, 54.02; H, 4.57; N, 12.64.
13. *1-methoxy-4-((E)-2-nitrovinyl) benzene (13)* (Kumar et al., 2012): IR (cm^−1^): 2959, 2924, 2360, 1598, 1506, 1304, 1252, 1167, 1019, 964, 798; 1H NMR (CDCl_3_,400 MHz): δ 3.87 (s, 3H), 6.95 (d, *J* = 8.4 Hz, 2H), 7.50 (d, *J* = 8.8 Hz, 2H), 7.52 (d, *J* = 13.6 Hz, 1H) 7.97 (d, *J* = 12.8 Hz, 1H); ESI-MS (m/z): 180 (M^+^+1); Anal. Calcd for (C_9_H_9_NO_3_): C, 60.33; H, 5.06; N, 7.82. Found: C, 60.34; H, 5.02; N, 7.84.
14. *1-methoxy-4-((E)-2-nitroprop-1-enyl) benzene (14)* (Yan et al., 2008): IR (cm^−1^): 2961, 2836, 1601, 1508, 1302, 1252, 1173, 1024, 823; ^1^H NMR (CDCl_3_,400 MHz): δ 2.46 (s, 3H), 3.85 (s, 3H), 6.97 (d, *J* = 8.8 Hz, 2H), 7.42 (d, *J* = 8.4 Hz, 2H), 8.06 (s, 1H); ESI-MS (m/z): 194 (M^+^+1); Anal. Calcd for (C_10_H_11_NO_3_): C, 62.17; H, 5.74; N, 7.25. Found: C, 62.19; H, 5.71; N, 7.27.
15. *1-methoxy-4-((E)-2-nitrobut-1-enyl) benzene (15)*: IR (cm^−1^): 3104, 2926, 2361, 1596, 1489, 1303, 1244, 1165, 1021, 964, 801; ^1^H NMR (CDCl_3_,300 MHz): δ 1.20 (t, *J* = 7.5 Hz, 3H), 281 (q, *J* = 7.5 Hz, 3H), 3.77 (s, 3H), 6.92 (d, *J* = 8.7 Hz, 2H), 7.33 (d, *J* = 8.7 Hz, 2H), 8.03 (s, 1H); ESI-MS (m/z): 208 (M^+^+1); Anal. Calcd for (C_11_H_13_NO_3_): C, 63.76; H, 6.32; N, 6.76. Found: C, 63.73; H, 6.35; N, 6.77.
16. *4-((E)-2-nitrovinyl) phenol (16)* (Kumar et al., 2012): IR (cm^−1^): 3742, 3134, 3016, 2362, 1695, 1509, 1303, 1228, 1155, 1017, 957, 802, 661; ^1^H NMR (CDCl_3_,400 MHz): δ 6.95 (d, *J* = 8.4 Hz, 2H), 7.49 (d, *J* = 8.8 Hz, 2H), 7.52 (d, *J* = 13.6 Hz, 1H), 7.97 (d, *J* = 13.6 Hz, 1H), 10.46 (brs, 1H); ESI-MS (m/z): 166 (M^+^+1); Anal. Calcd for (C_8_H_7_NO_3_): C, 58.18; H, 4.27; N, 8.48. Found: C, 58.21; H, 4.24; N, 8.52.
17. *4-((E)-2-nitroprop-1-enyl) phenol (17)* (Yan et al., 2008): IR (cm^−1^): 3648, 3147, 2956, 2361, 1684, 1513, 1285, 832; ^1^H NMR (CDCl3, 400 MHz): δ 2.46 (s, 3H), 5.23 (s, 1H), 6.92 (d, *J* = 8.4 Hz, 2H), 7.38 (d, *J* = 8.4 Hz, 2H), 8.06 (s, 1H); ^13^C NMR (CDCl_3_,100 MHz): 14.15, 116.05, 116.25, 123.07, 132.13, 132.43, 134.26, 144.64, 159.80; ESI-MS (m/z): 180 (M^+^+1); Anal. Calcd for (C_9_H_9_NO_3_): C, 60.33; H, 5.06; N, 7.82. Found: C, 60.31; H, 5.04; N, 7.86.
18. *4-((E)-2-nitrobut-1-enyl) phenol (18)*: IR (cm^−1^): 3745, 3125, 2983, 2363, 1691, 1516, 1302, 1252, 1173, 1024, 823; ^1^H NMR (CDCl3,400 MHz): δ 1.28 (t, *J* = 6.80 Hz, 3H), 2.88 (q, *J* = 6.4 Hz, 2H), 5.37 (s, 1H), 6.92 (d, *J* = 7.20 Hz, 2H), 7.36 (d, *J* = 7.20 Hz, 2H), 8.00 (s, 1H); ESI-MS (m/z): 194 (M^+^+1); Anal. Calcd for (C_10_H_11_NO_3_): C, 62.17; H, 5.74; N, 7.25. Found: C, 62.14; H, 5.76; N, 7.27.
19. *3-((E)-2-nitrovinyl) phenol (19)*: IR (cm^−1^): 3351, 3166, 3096, 2809, 2355, 2224, 1592, 1510, 1255, 1020, 959, 809; ^1^H NMR (CDCl_3_,400 MHz): δ 7.40–7.50 (m, 3H), 7.63 (d, *J* = 7.6 Hz, 1H), 7.71 (d, *J* = 8.0 Hz, 1H), 8.06 (d, *J* = 8.4, 1H); ESI-MS (m/z): 166 (M^+^+1); Calcd for (C_8_H_7_NO_3_): C, 58.18; H, 4.27; N, 8.48. Found: C, 58.16; H, 4.29; N, 8.49.
20. *3-((E)-2-nitroprop-1-enyl) phenol (20)* (Yan et al., 2008): IR (cm^−1^): 3271, 2967, 2927; ^1^H NMR (DMSO-D6,300 MHz): δ 2.36 (s, 3H), 5.30 (brs, 1H), 6.72–6.82 (m, 2H), 6.90 (d, *J* = 7.8 Hz, 1H), 7.23 (t, *J* = 8.1, 8.4 Hz, 1H), 7.94 (s, 1H); ESI-MS (m/z): 180 (M^+^+1); Anal. Calcd for (C_9_H_9_NO_3_): C, 60.33; H, 5.06; N, 7.82. Found: C, 60.36; H, 5.04; N, 7.79.
21. *3-((E)-2-nitrobut-1-enyl) phenol (21)*: IR (cm^−1^): 3741, 3101, 3027, 2923, 2855, 1694, 1521, 1464, 1024, 878, 797; ^1^H NMR (CDCl_3_,400 MHz): δ 1.20 (t, *J* = 7.20 Hz, 3H), 2.79 (q, *J* = 7.20 Hz, 2H), 5.12 (brs, 1H), 6.82–6.84 (m, 2H), 6.92 (d, *J* = 7.60 Hz, 1H), 7.25 (t, *J* = 7.60 Hz, 1H), 7.89 (s, 1H); ESI-MS (m/z): 194 (M^+^+1); Anal. Calcd for (C_10_H_11_NO_3_): C, 62.17; H, 5.74; N, 7.25. Found: C, 62.20; H, 5.75; N, 7.23.
22. *1,2-dimethoxy-4-((E)-2-nitrovinyl) benzene (22)* (Milhazes et al., 2006): IR (cm^−1^): 3122, 2357, 1691, 1499, 1335, 1222, 1132, 1009, 967, 798; ^1^H NMR (DMSO-D6,300 MHz): δ 3.81 (s, 3H), 3.83 (s, 3H), 7.05 (d, *J* = 8.40 Hz, 1H), 7.43 (dd, *J* = 8.4, 1.8 Hz, 1H), 7.49 (s, 1H), 8.06 (d, *J* = 13.5 Hz, 1H), 8.22 (d, *J* = 13.5 Hz, 1H); ^13^C NMR (CDCl_3_,100 MHz): 56.03, 56.10, 110.27, 111.36, 122.82, 124.66, 135.18, 139.36, 149.57, 152.83; ESI-MS (m/z): 210 (M^+^+1); ESI-MS (m/z): 224 (M^+^+1); Anal. Calcd for (C_10_H_11_NO_4_): C, 57.41; H, 5.30; N, 6.70. Found: C, 57.42; H, 5.33; N, 6.68.
23. *1,2-dimethoxy-4-((E)-2-nitroprop-1-enyl) benzene (23)* (Milhazes et al., 2006): IR (cm^−1^): 2959, 2929, 2840, 1650, 1592, 1509, 1448, 1307, 1257, 1136, 1017, 808; ^1^H NMR (DMSO-D6,300 MHz): δ 2.42 (s, 3H), 3.81 (s, 3H), 3.84 (s, 3H), 7.02–7.05 (m, 2H), 7.18 (dd, *J* = 8.4, 1.8 Hz, 1H), 8.16 (s, 1H); ESI-MS (m/z): 224 (M^+^+1); Anal. Calcd for (C_11_H_13_NO_4_): C, 59.19; H, 5.87; N, 6.27. Found: C, 59.22; H, 5.89; N, 6.21.
24. *1,2-dimethoxy-4-((E)-2-nitrobut-1-enyl) benzene (24)*: IR (cm^−1^): 3113, 2889, 1601, 1486, 1300, 1082, 960, 868, 819, 679; ^1^H NMR (DMSO-D6,300 MHz): δ 1.30 (t, *J* = 7.5 Hz, 3H), 2.91 (q, *J* = 7.5 Hz, 2H), 3.94 (s, 3H), 3.97 (s, 3H), 6.93–7.00 (m, 2H), 7.09 (dd, *J* = 8.4, 1.8 Hz, 1H), 8.01 (s, 1H); ESI-MS (m/z): 238 (M^+^+1); Anal. Calcd for (C_12_H_15_NO_4_): C, 60.75; H, 6.37; N, 5.90. Found: C, 60.71; H, 6.40; N, 5.93.
25. *4-((E)-1-nitroprop-1-en-2-yl)phenol (25):* IR (cm^−1^): 3289, 3066, 2964, 1655, 1567, 1349, 1268, 1207, 1155, 1097, 950, 808, 652; ^1^H NMR (CDCl_3_,400 MHz): δ 2.58 (s, 3H), 6.95 (d, *J* = 8.8 Hz, 2H), 7.81 (s, 1H), 7.91(d, *J* = 8.8 Hz, 2H); ESI-MS (m/z): 180 (M^+^+1); Anal. Calcd for (C_9_H_9_NO_3_): C, 60.33; H, 5.06; N, 7.82. Found: C, 60.29; H, 5.11; N, 7.83.
26. *3-((E)-3-nitropent-2-en-2-yl)phenol (26)*: IR (cm^−1^): 3740, 3096, 3051, 3003, 1691, 1519, 1214, 987, 873, 792; ^1^H NMR (DMSO-D_6_, 400 MHz): δ 1.18 (t, *J* = 6.8 Hz, 3H), 2.38 (S, 3H), 2.57 (m, 2H), 6.97 (d, *J* = 7.2 Hz, 1H), 7.23–7.25 (m, 2H), 7.33 (t, *J* = 7.6 Hz, 1H), 9.76 (s, 1H); ^13^C NMR (DMSO-D6, 100 MHz): 14.48, 20.11, 26.15, 113.69, 118.22, 130.20, 136.24, 157.89, 162.76, 168.80, 171.92; ESI-MS (m/z): 208 (M^+^+1); Anal. Calcd for (C_11_H_13_NO_3_): C, 63.76; H, 6.32; N, 6.76. Found: C, 63.79; H, 6.26; N, 6.78.
27. *2-ethoxy-6-((E)-2-nitrovinyl) phenol (27)*: IR (cm^−1^): 3372, 2973, 2359, 1594, 1483, 1336, 1272, 1054, 810; ^1^H NMR (CDCl_3_,400 MHz): δ 1.49 (t, *J* = 7.2 Hz, 3H), 4.16 (q, *J* = 7.2 Hz, 2H), 6.10 (s, 1H), 6.96–6.98 (m, 2H), 7.12 (dd, *J* = 8.4, 1.6 Hz, 1H), 7.49 (d, *J* = 13.2 Hz, 1H), 7.94 (d, *J* = 13.6 Hz, 1H); ESI-MS (m/z): 210 (M^+^+1); Anal. Calcd for (C_10_H_11_NO_4_): C, 57.41; H, 5.30; N, 6.70. Found: C, 57.43; H, 5.28; N, 6.72.
28. *3-((E)-2-nitrovinyl)-1H-indole (28)*: IR (cm^−1^): 3398, 3100, 2921, 2356, 1608, 1470, 1307, 1229, 1098, 745; ^1^H NMR (CDCl_3_,400 MHz): δ 7.41–7.46 (m, 2H), 7.56-7.58 (m, 1H), 7.77 (d, *J* = 3.2 Hz, 1H), 7.88–7.91 (m, 2H), 8.38 (d, *J* = 13.2 Hz, 1H), 8.87 (brs, 1H); ESI-MS (m/z): 189 (M^+^+1); Anal. Calcd for (C_10_H_8_N_2_O_2_): C, 63.82; H, 4.28; N, 14.89. Found: C, 63.88; H, 4.32; N, 14.82.
29. *3-((E)-2-nitroprop-1-enyl)-1H-indole (29)* (Yan et al., 2008): IR (cm^−1^): 3420, 3113, 2883, 2359, 1698, 1623, 1519, 1395, 1262, 1212, 1096, 964, 856, 742; ^1^H NMR (DMSO-D6,300 MHz): δ 2.40 (s, 3H), 7.18–7.28 (m, 2H), 7.51 (d, *J* = 7.8 Hz, 1H), 7.83 (d, *J* = 7.5 Hz, 1H), 8.01 (s, 1H), 8.46 (s, 1H), 12.20 (brs, 1H); ^13^C NMR (DMSO-D_6_,100 MHz): 15.07, 108.71, 112.84, 118.69, 121.48, 123.40, 127.08, 127.98, 130.50, 136.64, 141.45; ESI-MS (m/z): 203 (M^+^+1); Anal. Calcd for (C_11_H_10_N_2_O_2_): C, 65.34; H, 4.98; N, 13.85. Found: C, 65.38; H, 5.02; N, 13.82.
30. *3-((E)-2-nitrobut-1-enyl)-1H-indole (30)*: IR (cm^−1^): 3307, 2969, 2936, 2354, 1618, 1417, 1256, 1205, 1059, 1004, 934, 731, 683; ^1^H NMR (CDCl_3_, 400 MHz): δ 1.31 (t, *J* = 7.2 Hz, 3H), 2.98 (q, *J* = 7.2 Hz, 2H), 7.26-7.35 (m, 2H), 7.47 (d, *J* = 8.0 Hz, 1H), 7.58 (d, *J* = 2.0 Hz, 1H), 7.82 (d, *J* = 7.60 Hz, 1H), 8.47 (s, 1H), 8.83 (brs, 1H); ESI-MS (m/z): 217 (M^+^+1); Anal. Calcd for (C_12_H_12_N_2_O_2_): C, 66.65; H, 5.59; N, 12.96. Found: C, 66.68; H, 5.62; N, 13.01.

### Microbial strains

One reference each of the following species was used for their *in vitro* susceptibility to β-nitrostyrene in this study: *Staphylococcus aureus* ATCC 29213, *Staphylococcus epidermidis* ATCC 14990, *Escherichia coli* ATCC 25922, *Pseudomonas aeruginosa* ATCC 27853, *Candida albicans* ATCC 90028, *Candida glabrata* ATCC 90030, *Aspergillus fumigatus* ATCC 16424 and *Aspergillus niger* ATCC 16404. These strains were procured from the American Type Culture Collection (ATCC, Manassas, VA, USA).

### Leishmanial parasite culture and maintenance

*L. donovani* parasites (MHOM/IN/83/AG83) were maintained *in vivo* in BALB/c mice. Promastigotes were routinely cultured at 22°C in medium M199 supplemented with 10% heat inactivated fetal bovine serum (FBS, HiMedia Laboratories, Mumbai, India), penicillin 100 (IU/ml), streptomycin (100 μg/ml). Log phase promastigotes were sub-cultured every 72–96 h, the inoculum being 2 × 10^6^ cells/ml (Afrin et al., 2002).

### Cell line culture

Murine macrophage cell line, RAW264.7 was grown at 37°C in medium RPMI-1640 (pH 7.4, Sigma-Aldrich, St. Louis, MO, USA) supplemented with 10% heat-inactivated FBS for 48 to 72 h in a humidified atmosphere of 5% CO_2_ and sub-cultured in fresh RPMI-1640 medium at an average density 2 × 10^5^ cells/ml (Islamuddin et al., 2014b).

### Antimicrobial susceptibility testing assays

The antibacterial and antifungal activities of the β-nitrostyrenes were performed by broth microdilution methods as per the guidelines of Clinical and Laboratory Standard Institute (Wayne, 1998, 2008a,b,c) by using Mueller-Hinton broth (MHB; Becton-Dickinson, Cockeysville, MD, USA) supplemented with calcium (25 mg/L) and magnesium (12.5 mg/L) for bacterial strains while as RPMI 1640 medium with _L_-glutamine and without sodium bicarbonate, buffered to pH 7.0 with 0.165 M 3-(*N*-morpholino) propanesulfonic acid (MOPS; both from Sigma) was used for fungal strains. Stock solutions were prepared in 100% dimethyl sulfoxide (DMSO; Sigma; distilled water was used for ciprofloxacin), with a final DMSO concentration of 1% (vol. per vol.) and two-fold serial dilutions were prepared in media in amounts of 100 μL per well in 96-well U-bottom microtiter plates (Tarsons, Kolkata, India) to yield twice the final concentration required for testing, which ranged from 64 to 0.12 μg/mL for β-nitrostyrenes and 16 to 0.03 μg/mL for ciprofloxacin as well as amphotericin B. Stock inoculum suspensions of the bacteria, *Candida* species and *Aspergillus* species were prepared in sterile normal saline (0.85%) containing 0.05% polysorbate 20 (NST) from overnight (3 to 5-day old for *Aspergillus* species) strains grown on Trypticase soy agar (TSA; Becton-Dickinson) and potato dextrose agar (Difco Laboratories, Detroit, MI, USA) respectively at 35°C. Inocula were verified for each assay by plating onto agar plates for colony enumeration. These suspensions were further diluted in the respective test medium and a 100 μl volume of this diluted inoculum was added to each well of the plate, resulting in a final inoculum concentration of approximately 5 × 10^5^ CFU/mL for bacter1ia, (Wayne, 2008a) approximately 2.5 × 10^4^ CFU/ml for *Candida* species (Pfaller et al., 2004) while as 0.4 × 10^4^ to 5 × 10^4^ CFU/mL for *Aspergillus* species (Wayne, 2008c). Untreated growth control, vehicle control (DMSO) and sterility control wells were included for each isolate tested. Ciprofloxacin and amphotericin B served as the standard drug controls for bacterial and fungal strains, respectively. The microtiter plates were incubated at 35°C for 24 h for bacterial strains and 48 h for fungal strains. The plates were read visually and the minimum inhibitory concentration (MIC) was defined as the lowest concentration of test sample that prevented visible growth with respect to the growth control.

The minimum bactericidal concentration (MBC) and minimum fungicidal concentration (MFC) were determined by plating 100 μl volumes on agar plates from the wells showing no visible growth (clear MIC well). The plates were incubated as described above in MIC measurements. The minimum concentration of β-nitrostyrenes and drugs that showed ≥99.9% reduction of the original inoculums was recorded as the MBC as well as MFC9 (Wayne, 1998; Pfaller et al., 2004). All experiments were conducted twice in duplicates on separate occasions with freshly prepared inoculums and stock solutions.

### Anti-leishmanial activity

Nitrostyrine series were screened for their antileishmanial activity against *L. donovani* promastigotes *in vitro*. Promastigotes of *L. donovani* (2 × 10^6^ cells/mL) in M199 medium were incubated at 22°C for 72 h with each compound at a concentration of 100 μM. Amphotericin B (AmB) served as reference drug, 0.2% DMSO was used as solvent control. Parasites with media alone were taken as control. Parasite viability was determined microscopically after 72 h (Islamuddin et al., 2014b).

### Dose-dependent anti-promastigote activity and determination of IC_50_

Promastigotes at density of 2 × 10^6^ cells/mL were incubated in the absence and or presence of most active series of nitrostyrine (compounds 9, 12, 14, and 18) at serial four-fold dilutions starting at 25 μM (25, 6.25, 1.56, 0.39, 0.097, 0.024, and 0.008 μM) for 3 days at 22°C. AmB was used as a standard antileishmanial drug control. The cell viability was evaluated by microscopically and the mean percentage viability was calculated as follows: Mean cell number of treated parasites / Mean cell number of untreated parasites × 100. The 50 and 90% inhibitory concentration (IC_50_ and IC_90_) i.e., the concentration of drugs that decreased the cell growth by 50 and 90% respectively was determined by graphical extrapolation after plotting the graph of % viability vs. concentration of drug (Islamuddin et al., 2014b).

### Leishmanicidal assay

To confirm the leishmanicidal effect of compounds 9, 12, 14, and 18, treated and untreated parasites (1 μM) after 72 h of incubation, were washed two times with fresh incomplete medium and finally resuspended in complete media, transferred back to 22°C and allowed to grow for 72 h. The viability of the parasites was determined microscopically (Afrin et al., 2001).

### Determination of promastigote cellular morphology

Changes in the cellular morphology of *Leishmania* parasites as a result of compounds 9, 12, 14, and 18 treatment was observed microscopically. Briefly, the promastigotes (2 × 10^6^ cells/mL) were incubated in the absence or presence of compounds and AmB for 72 h at a concentration of 1 μM and observed under 40 × objective of a phase-contrast microscope. At least 20 microscopic fields were observed for each sample. Data were recorded by using NIS-Elements imaging software (Paris et al., 2004).

### Anti-amastigote activity and nitrite production in an *Ex-vivo* macrophage model

Macrophages from the peritonial cavity of BALB/c mice (1 × 10^6^ cellsml^−1^) were allowed to adhere to round glass coverslips in a 24 well tissue culture plate and incubated for 12 h at 37°C in a CO_2_ incubator. After removal of the non-adherent macrophages, the cells were infected with stationary phase promastigotes using a cell to parasite ratio of 1:10 and incubated at 37°C for 24 h. The non-phagocytosed parasites were removed by washing, and the infected macrophages incubated with the test compounds 9, 12, 14, and 18 (0–25 μM) for an additional 48 h. The coverslips were fixed in methanol and giemsa-stained for microscopic evaluation (100X) of amastigote infectivity. Number of amastigotes per 100 macrophages were enumerated and the concentration that decreased amastigotes by 50% (IC_50_) was determined (Wong et al., 2012; Islamuddin et al., 2014a).

The supernatant from control *L. donovani* infected and treated macrophages were analyzed for nitrite content by the Griess reaction. In brief, equal volume of Griess reagent [1%sulphanilamide and 0.1% N-(1-naphthyl) ethylenediaminedihydrochloride in 5% phosphoric acid] was added to the culture supernatant and the plates were incubated for 10 min at room temperature using sodium nitrite as a standard. The absorbance at 550 nm was measured and the concentration of nitrite was calculated by a linear regression analyzing the standard curve generated with sodium nitrite (Varela et al., 2012).

### *Ex vivo* cytotoxicity assay

To evaluate the adverse toxicity of test compounds (9, 12, 14, and 18) on mammalian cells, the murine macrophage cell line (RAW264.7) cultured in RPMI 1640 medium were exposed to increasing concentrations of test compounds (5–50 μM) at 37°C, 5% CO_2_ for 48 h. AmB as the reference drug, and macrophages without any treatment were taken as control. The percentage of cell viability was evaluated by MTT (3-{4, 5-dimethylthiazol-2-yl}-2, 5-diphenyltetrazoliumbromide) assay (Das et al., 2008).

### Statistical analysis

*In vitro* antileishmanial activity was expressed as IC_50_ by linear regression analysis. Values are mean ± SE of samples in triplicate from at least three independent experiments. Statistical analysis was performed using ANOVA followed by Dunnett's test. ^*^P < 0.05, ^**^P < 0.01 and ^***^P < 0.001 compared with the untreated control group were considered significant.

## Results

### Chemistry

**Scheme 1:**
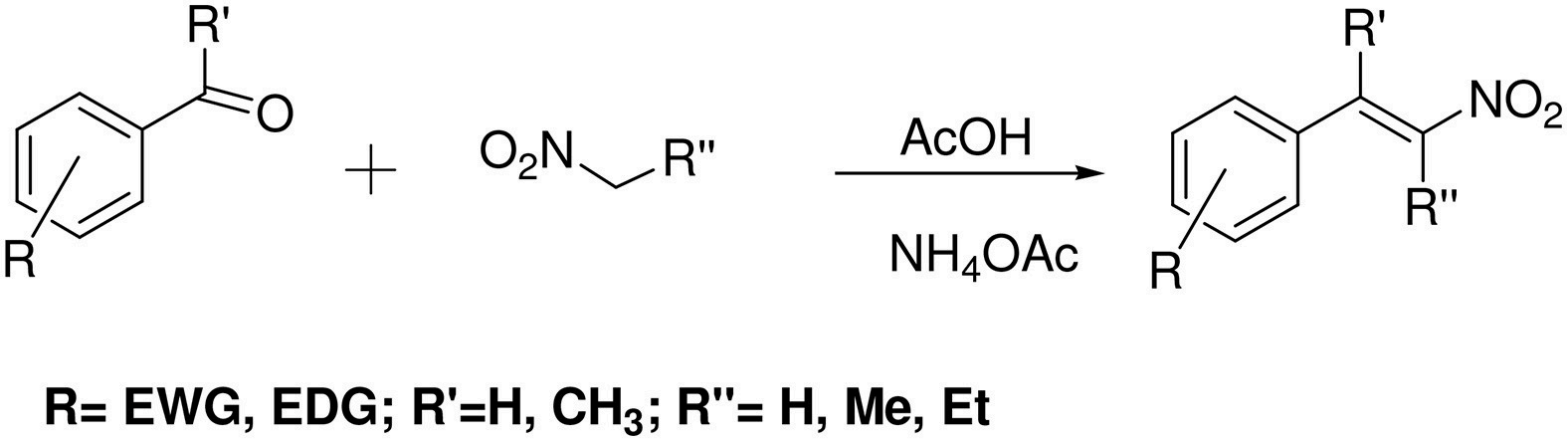
**Synthesis**.

β-Nitrostyrene derivatives were prepared by a widely used Henry reaction (Gairaud and Lappin, 1953; Huitric et al., 1956; Milhazes et al., 2006; by a nitroaldol condensation reaction-Henry reaction). The compounds were synthesized by the reaction of aromatic aldehydes/ ketones with nitromethane/nitroethane/nitropropane in presence of ammonium acetate in acetic acid. These experimental conditions led to moderate to high yields of the target molecules. The final products were identified by NMR (^1^H NMR), FT-IR and MS-EI spectroscopic techniques. These compounds have been assigned as *E*-isomers based on the coupling constants and the existing literature (Scheme 1).

A series of β-nitrostyrene derivatives were synthesized (Table 1) in order to study the influence of different aromatic substitution patterns and alkyl substitutions at α/β positions on the antimicrobial/anti-leishmanial activities. Melting points and NMR data of the synthesized β-nitrostyrenes were consistent with the literature values (Gairaud and Lappin, 1953; Somasekhara et al., 1971; Bose et al., 1992; Wang and Wang, 2002; Al-Zereini et al., 2007; Yan et al., 2008; Kumar et al., 2012; Manna et al., 2013; Cornell et al., 2014).

**Table 1:** **Synthetic library of β-nitrostyrenes**.

**Compound**	**Basic moiety**	**R**	**R'**	**R“**	**Yields^a^ (%)**	**M.P.(°C)**	**M.P.reference**
1	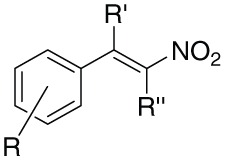	4-Cl	H	H	81	112–114	113–114
2		4-Cl	H	Me	82	88–90	89–91
3		4-Cl	H	Et	80	105–107	
4		4-NMe2	H	H	91	186–188	186–188
5		4-NMe_2_	H	Me	96	90–91	
6		4-NMe_2_	H	Et	92	77–79	
7		4-NO_2_	H	H	90	94–96	94–96
8		4-NO_2_	H	Me	89	114–116	114–115
9		4-NO_2_	H	Et	81	104–106	103.5–104.5
10		3-NO_2_	H	H	79	124–126	125
11		3-NO_2_	H	Me	81	80–82	
12		3-NO_2_	H	Et	84	84–86	
13		4-OMe	H	H	89	86–88	85–87
14		4-OMe	H	Me	88	44–46	44–45
15		4-OMe	H	Et	73	Oil	Oil
16		4-OH	H	H	76	168–169	167–171^b^
17		4-OH	H	Me	72	122–124	124–125
18		4-OH	H	Et	71	60–62	
19		3-OH	H	H	79	138–140	136–140^b^
20		3-OH	H	Me	81	96–98	96–98
21		3-OH	H	Et	79	82–84	
22		3,4-dimethoxy	H	H	88	140–142	142–144
23		3,4-dimethoxy	H	Me	89	70–72	71–72
24		3,4-dimethoxy	H	Et	91	78–80	78–79
25		4-OH	Me	H	77	88–90	
26		3-OH	Me	Et	74	138–140	
27		2-OH,3-OEt	H	H	87	128–130	
28	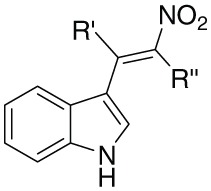	-	H	H	89	134–136	
29		-	H	Me	91	182–184	
30		-	H	Et	86	64–66	

a *Isolated yields*.

b *^b^Melting points of compounds from sigma aldrich*.

### *In vitro* antimicrobial susceptibility results

All the above synthesized β-nitrostyrenes were tested for their *in vitro* antibacterial as well as antifungal activities against two references of strains of each (total eight strains) selected gram-positive bacteria, gram-negative bacteria, *Candida* and *Aspergillus* species by broth microdilution method following Clinical and Laboratory Standards Institute (CLSI) guidelines. The results are tabulated in Tables 2, 3.

**Table 2:** ***In vitro* antibacteriall activity of β-Nitrostyrene**.

**Test compounds**	**Antibacterial activity-MIC in (**μ**g/mL)**							
	**Sa ATCC 29213**		**Se ATCC 14990**		**Ec ATCC 25922**		**Pa ATCC 27853**	
	**MIC**	**MBC**	**MIC**	**MBC**	**MIC**	**MBC**	**MIC**	**MBC**
Cipro	0.25	0.5	0.25	0.25	<0.03	<0.03	0.25	1.0
1	32	32	16	16	32	32	32	64
2	>64	>64	32	32	>64	>64	>64	>64
3	4	4	2	2	>64	>64	>64	>64
4	>64	>64	>64	>64	>64	>64	>64	>64
5	>64	>64	>64	>64	>64	>64	>64	>64
6	>64	>64	>64	>64	>64	>64	>64	>64
7	8	8	4	4	16	32	16	32
8	16	32	16	16	64	>64	64	>64
9	8	8	4	4	16	32	16	32
10	NT	NT	NT	NT	NT	NT	NT	NT
11	8	16	8	8	32	64	64	>64
12	0.5	0.5	0.25	0.25	16	16	16	16
13	64	64	32	32	>64	>64	>64	>64
14	8	8	4	4	64	>64	>64	>64
15	16	32	16	16	>64	>64	>64	>64
16	64	>64	64	64	64	>64	>64	>64
17	4	4	2	2	16	16	16	32
18	8	16^*^	8	8	>64	>64	>64	>64
19	>64	>64	64	>64	>64	>64	>64	>64
20	2	4	2	2	16	16	16	32
21	2	4	2	2	32	64	32	64
22	>64	>64	64	64	>64	>64	>64	>64
23	8	16	8	8	>64	>64	>64	>64
24	NT	NT	NT	NT	NT	NT	NT	NT
25	NT	NT	NT	NT	NT	NT	NT	NT
26	8	16	8	8	>64	>64	>64	>64
27	32	32	16	16	>64	>64	>64	>64
28	64	>64	64	64	64	>64	>64	>64
29	16	16	8	8	>64	>64	>64	>64
30	16	16	8	8	64	>64	>64	>64

*NT, not tested; Sa, Staphylococcus aureus; Se, Staphylococcus epidermidis; Ec, Escherichia coli; Pa, Pseudomonas aeruginosa; MIC, minimum inhibitory concentration; MBC, minimum bactericidal concentration; Ciprofloxacin served as drug control for bacterial strains. The results are representative of two separate experiments performed in duplicate and similar results were observed each time*.

**Table 3:** ***In vitro* antifungal activity of β-Nitrostyrenes**.

**Test compounds**	**Antifungal activity-MIC in (**μ**g/mL)**							
	**Ca ATCC 90028**		**Cg ATCC 90030**		**Af ATCC 16424**		**An ATCC 16404**	
	**MIC**	**MFC**	**MIC**	**MFC**	**MIC**	**MFC**	**MIC**	**MFC**
AmB	0.5	0.5	0.5	0.5	0.5	1.0	1.0	1.0
1	32	32	4	4	8	8	4	4
2	>64	>64	32	32	>64	>64	64	>64
3	>64	>64	>64	>64	32	64	32	32
4	>64	>64	32	32	64	>64	64	>64
5	>64	>64	32	32	>64	>64	64	>64
6	>64	>64	32	32	>64	>64	64	>64
7	4	4	4	4	4	4	2	2
8	4	4	1	1	4	4	2	2
9	8	8	0.25	0.25	8	8	4	4
10	NT	NT	NT	NT	NT	NT	NT	NT
11	NT	NT	NT	NT	NT	NT	NT	NT
12	2	2	0.25	0.25	1	1	0.5	0.5
13	64	64	32	32	64	>64	64	64
14	32	32	8	8	8	8	4	4
15	32	32	16	16	8	16	8	8
16	32	32	16	16	16	16	8	16
17	8	8	8	8	8	8	4	4
18	16	16	8	8	8	16	4	8
19	64	>64	16	16	64	>64	64	64
20	16	16	8	8	8	8	4	4
21	32	32	16	16	8	8	4	8
22	64	64	16	16	32	64	32	32
23	32	32	4	4	16	16	8	8
24	NT	NT	NT	NT	NT	NT	NT	NT
25	NT	NT	NT	NT	NT	NT	NT	NT
26	>64	>64	>64	>64	>64	>64	64	>64
27	32	32	16	16	16	16	8	8
28	32	32	16	16	16	16	8	16
29	64	64	8	8	16	16	8	8
30	>64	>64	32	64	64	64	32	32

*NT, not tested; Ca, Candida albicans; Cg, Candida glabrata; Af, Aspergillus fumigatus; An, Aspergillus niger; MIC, minimum inhibitory concentration; MFC, minimum fungicidal concentration. Aamphotericin B was used as drug control for fungal strains. The results are representative of two separate experiments performed in duplicate and similar results were observed each time*.

Compound 12 exhibited broad-spectrum and potent activity against a variety of selected pathogenic strains, with the MIC ranging from 0.25 to 0.5 μg/mL for gram-positive bacteria (*S. aureus* and *S. epidermidis*), 16 μg/mL for both strains of *E. coli* and *P. aeruginosa* (Gram-negative bacteria), 0.25 to 2.0 μg/mL for *Candida* species and 0.5 to 1.0 μg/mL for *Aspergillus* species, respectively.

It was observed that all tested strains were susceptible to compounds 3, 7, 9, 17, 18, 20, and 21. These compounds exhibited the minimum inhibitory concentration (MIC) ranging from 2 to 32 μg/mL for *S. aureus*, 2–16 μg/mL for *S. epidermidis*, 16–32 μg/mL for both strains of *E. coli* and *P. aeruginosa*, 4–32 μg/mL for *C. albicans*, 0.25–16 μg/mL for *C. glabrata*, 4–8 μg/mL for *A. fumigatus* and 2–4 μg/mL for *A. niger*, respectively.

The minimum bactericidal concentrations (MBCs) were found to be either same or only two-fold greater than MICs and the minimum fungicidal concentrations (MFCs) were also found to be either same or only two-fold greaterthan MICs, which indicates that β-nitrostyrenes have microbicidal effect against all tested species. Among all the fungal species tested, *C. glabrata* (MIC and MFC were 0.25 μg/mL for compound 9 as well as 12, respectively) was found to be the most susceptible species. One percent DMSO (vehicle control) had no inhibitory effect on the growth of tested fungal species, when compared with growth control.

### Anti-leishmanial effect

*L. donovani* promastigotes were treated with β-nitrostyrenes (100 μM) and their viability ascertained under phase contrast microscope (40X). All the parasites were found to be dead when treated with compounds 7, 8, 9, 11, 12, 14, 17, 18, 20, 21, 23; while few parasites survived with rest of the compounds after 72 h of incubation.

### IC_50_ and IC_90_ values of selected compounds against *L. donovani* promastigotes

**Figure 2:**
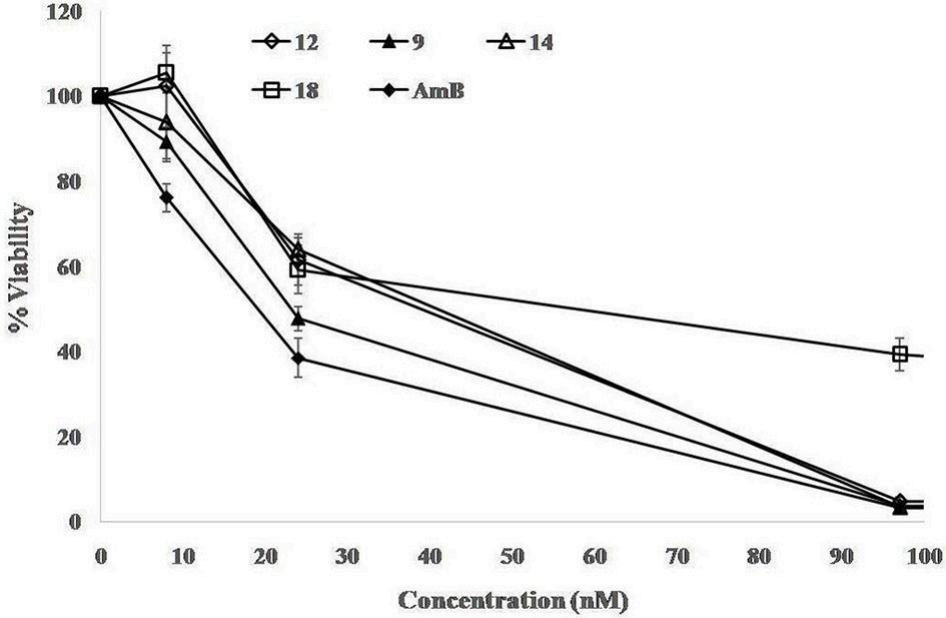
**Estimation of IC_50_ and IC_90_ of Compounds 9, 12, 14, and 18 against promastigotes**. Parasites (2 × 10^6^ cells/mL) were incubated with serial four-fold dilutions of test compounds (starting at 25 μM) for 72 h and viability was determined microscopically, as described in methods. Each point or bar corresponds to the mean ± SE of triplicate samples and is representative of one of three independent experiments.

The compounds (7, 8, 9, 11, 12, 14, 17, 18, 20, 21, and 23) with potent anti-leishmanial activity (from the above anti-leishmanial susceptibility results) were further evaluated for their 50 and 90% growth inhibitory effect. Treatment with these compounds demonstrated a dose dependent killing effect on the promastigotes. Fifty percent inhibitory concentration of the compounds is depicted in the Table 4 where compounds 9, 12, 14, and 18 were found to be more potent, exhibiting IC_50_ in nM range. The 50 and 90% inhibitory concentration of the highly potent compounds (9, 12, 14, and 18) is represented in Figure 2. The established anti-leishmanial drug AmB served as a positive drug control, showed a similar trend in dose dependent parasite killing with IC_50_ and IC_90_ values at 19.60 ± 1.71 and 82.56 ± 1.36 nM respectively. Parasite viability was not affected by DMSO (0.2%, data not shown) used as solvent control.

**Table 4:** ***In vitro* antileishmanial activities of β-Nitrostyrenes 9, 12, 14, 18, and AmB against *L. donovani* promastigotes and intracellular amastigotes**.

**S.No**	**Compound**	***L. donovani promastigotes* IC90 ± SEM (nM)^a^**	***L. donovani* promastigotes IC50 ± SEM (nM)^a^**	***L. donovani amastigotes* IC50 ± SEM (nM)^a^**
1	9	90.16 ± 1.11	23.40 ± 0.71	30.5 ± 3.42
2	12	86.46 ± 0.21	37.83 ± 3.74	21.46 ± 0.96
3	14	89.00 ± 0.52	40.50 ± 1.47	26.43 ± 2.71
4	18	320.50 ± 3.65	55.66 ± 2.84	61.63 ± 8.02
5	AmB	82.56 ± 1.36	19.60 ± 1.71	27.83 ± 3.26

*SEM, Standard Error of the Mean*;

a *Mean from three determinations*.

### Leishmanicidal effect of the treated compounds

**Figure 3:**
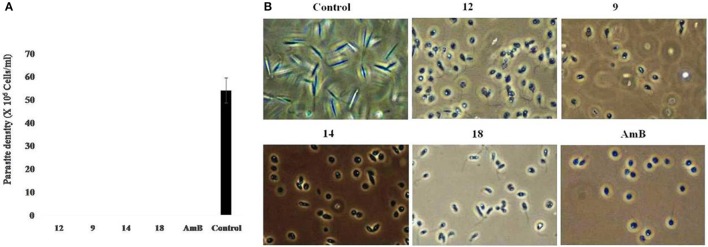
**(A)** Leishmanicidal effects of treated promastigotes with compounds 9, 12, 14, and 18 revealed no reversion of growth. Each point or bar corresponds to the mean ± SE of triplicate samples and is representative of one of three independent experiments. **(B)** Analysis of cellular morphology of promastigotes treated with compounds 9, 12, 14, 18 and ampotericin B. Exponential-phase promastigotes (2 × 10^6^ cells/mL) were incubated with 1μM of compounds 9, 12, 14, 18 for 72 h and analyzed by light microscopy (400 magnification), as described in Methods.

To determine the lethal effects of compounds 9, 12, 14, and 18 on promastigotes, treated and untreated parasites after 72 h were washed and resuspended in fresh media. After an additional 72 h in culture, the parasites did not revert back to their normal morphology and still appeared dead in case of prior incubation with 9, 12, 14, and 18, as also observed in positive drug control (AmB), confirming their leishmanicidal effects. Untreated control promastigotes reverted to late log phase (Figure 3A).

### Cellular morphological alterations in *L. donovani* promastigotes

Microscopic assessment of promastigotes upon treatment with compounds 9, 12, 14, and 18 at 1 μM revealed that the parasites altered to an ovoid shape with cell shrinkage, loss of flagella, cytoplasmic condensation, resulting in complete circularization of the promastigotes and substantial reduction in size compared to the untreated control. These changes, typical of programmed cell death (Figure 3B) were also observed in AmB treated parasites.

### Effect of test compounds (9, 12, 14, and 18) on *L. donovani* intracellular amastigote forms

**Figure 4:**
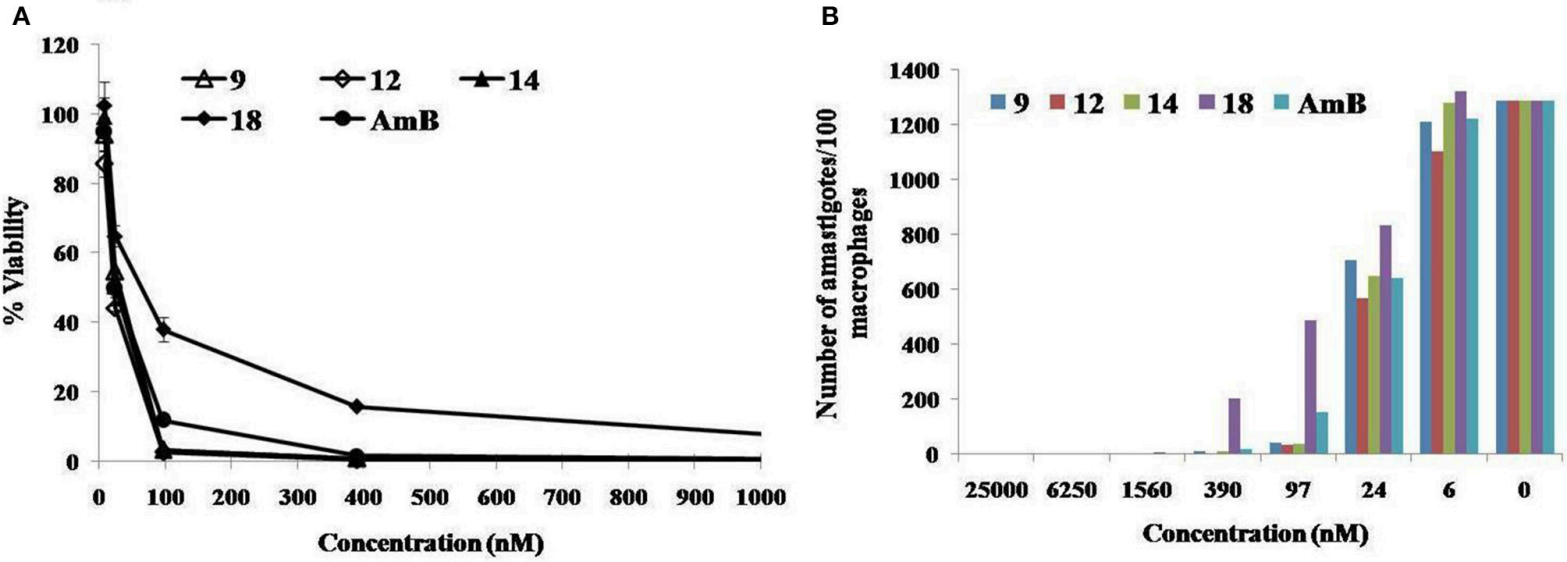
**Effects of β-nitrostyrenes (9, 12, 14, and 18) compounds on *L. donovani* intracellular amastigote forms**. After infection with promastigotes, peritoneal macrophages were incubated with serial four-fold dilutions of β-nitrostyrenes compounds (starting at 25000-0 nM) for 48 h and infection **(A)** as a percentage of control, **(B)** as number of amastigotes per 100 macrophages) was determined microscopically, as described in Methods. Each point corresponds to the mean ± SE of triplicate samples and is representative of one of three independent experiments.

Most active compounds against promostigotes were selected for the further studies against intracellurar amastigotes. Anti-amastigote activity of β-nitrostyrenes (9, 12, 14, and 18) on phagocytosed amastigotes within the macrophages was determined by giemsa stain method under microscopic observation. Compounds 9, 12, 14, and 18 demonstrated a high degree of activity with IC_50_ 30.5 ± 3.42, 21.46 ± 0.96, 26.43 ± 2.71 and 61.63 ± 8.02 nM respectively (Table 4) when compared to the standard AmB (27.83 ± 3.26 nM). Giemsa-stained micrographs of infected macrophages showed almost total clearance of the intracellular amastigotes at the highest dose of 1.56 μM, similar effect was observed with AmB (Figure 4).

### β-Nitrostyrenes (9, 12, 14, and 18) induced nitric oxide production in *Ex vivo* model

**Figure 5:**
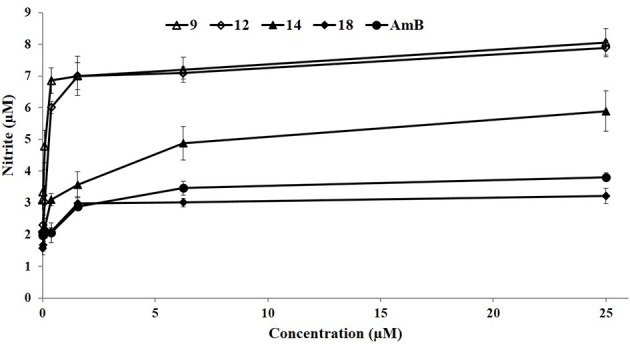
**β-Nitrostyrenes (9, 12, 14, and 18) induced nitric oxide production in ex *vivo* model**. Macrophages isolated from peritoneal cavity of BALB/c mice, infected with promastigotes and stimulated with β-nitrostyrenes (9, 12, 14, and 18) compounds at 37°C in a CO_2_ incubator with serial four-fold dilutions of test compounds (starting at 25–0 μM) for 48 h. Nitrite generation was determined by Gries methods, as described in Methods. Each point corresponds to the mean ± SE of triplicate samples and is representative of one of three independent experiments.

Nitric oxide (NO) levels were estimated in the culture supernatants of normal macrophages and infected macrophages. Infected macrophages demonstrated low basal levels of NO confirming the disease progression. NO level of 9, 12, 14, and 18 treated macrophages were estimated by using giess method at 0–25 μM concentrations for 48 h. A mild increase in NO production was observed with compounds 9 (8.05 μM) and 12 (7.89 μM) followed by compound 14 (5.89 μM), 18 (3.22 μM) and the standard AmB (3.81) at higher concentration (25 μM; Figure 5).

### Cytotoxicity of the test compounds (9, 12, 14, and 18) on RAW264.7 macrophage cell line

**Figure 6:**
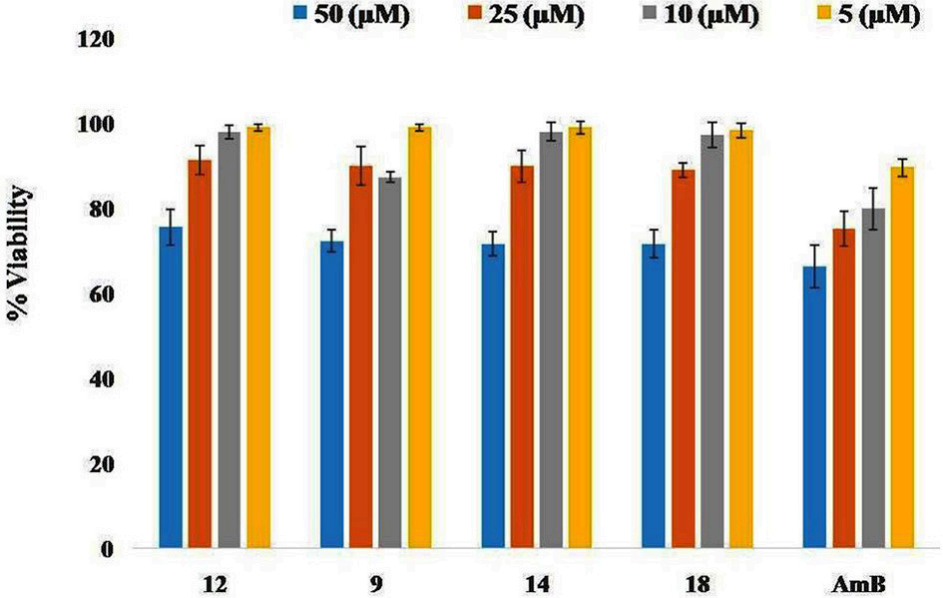
**Absence of adverse toxicity of test compounds 9, 12, 14, and 18 on macrophage cell line RAW264.7**. The cell lines were incubated for 48 h at 37°C in a CO_2_ incubator with increasing concentrations of test compounds or AmB and viability was ascertained. Each point or bar corresponds to the mean ± SE of triplicate samples and is representative of one of three independent experiments.

*In vitro* cytotoxicity assay was carried out with macrophage cell line RAW264.7 to investigate the side effects of the test compounds using AmB as a reference drug. The toxicity assay revealed that test compounds up to 10 μM had no adverse effects on the viability and morphology of the macrophages unlike the standard drug, AmB (Figure 6).

## Discussion

VL is considered as an opportunistic infection among immunocompromised patients and mainly treated with toxic pentavalent antimonials, second line drugs such as amphotericin B and pentamidine. Due to severe adverse and toxic profile of synthetic molecules, there is an essential requirement of new, safer and active principles from nature (Sundar et al., 2000). Hence natural products once again got the attention and several groups are working on the development of natural product based new anti-leishmanial agents (Barata et al., 2000; Rocha et al., 2005; Sharma et al., 2013; Islamuddin et al., 2014a,b; Jameel et al., 2014; Rottini et al., 2015). Over the years several antifungal and anticancer agents were screened for their anti-leishmanial potentials. Though there are some positive results obtained but they also suffered with acquired resistance and toxicity. Hence the search for new potential anti-leishmanial agents with better safety profiles from natural sources is being continued and is one of the core challenges in the current drug discovery.

**Figure 7:**
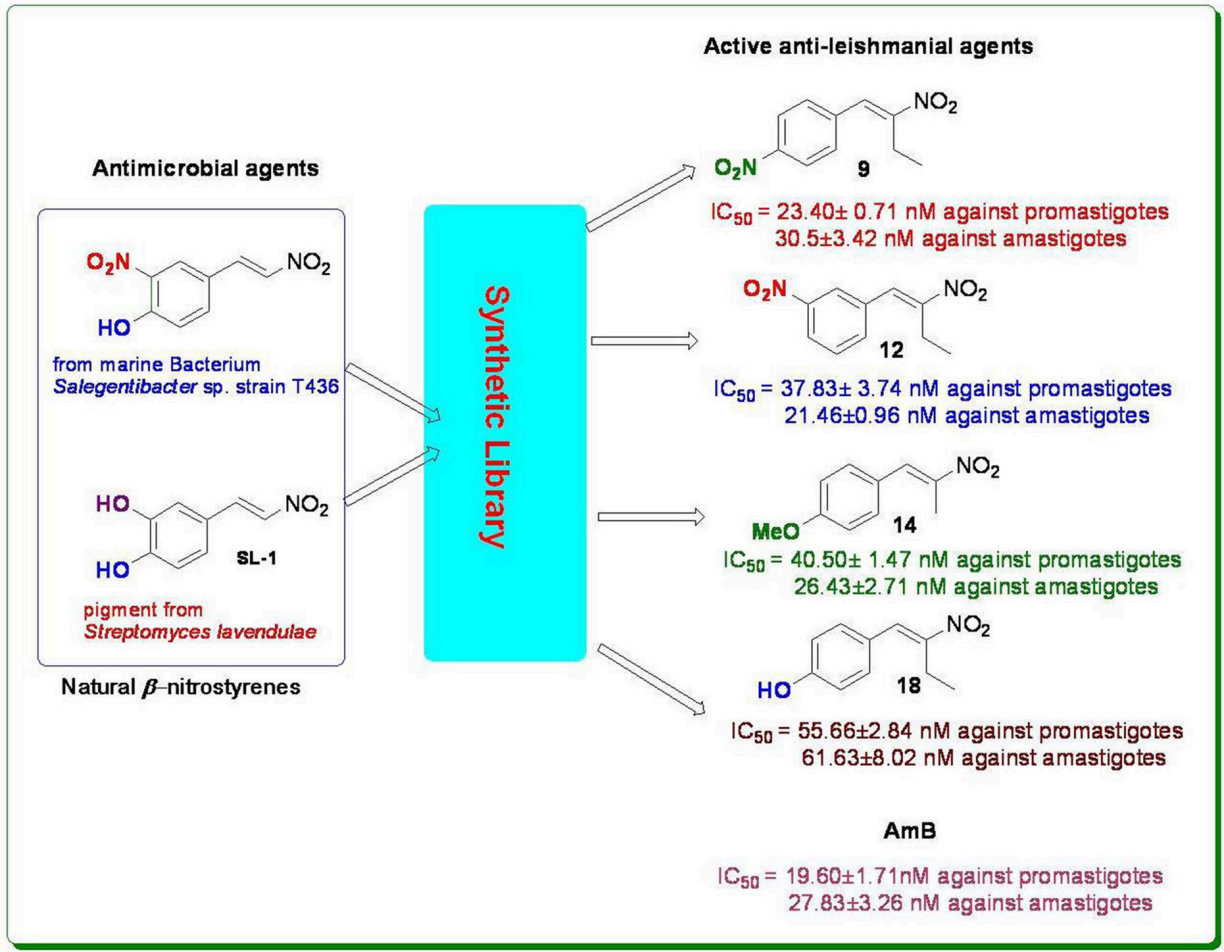
**Diversity oriented synthesis of natural products inspired β-nitrostyrene library**.

**Figure 8:**
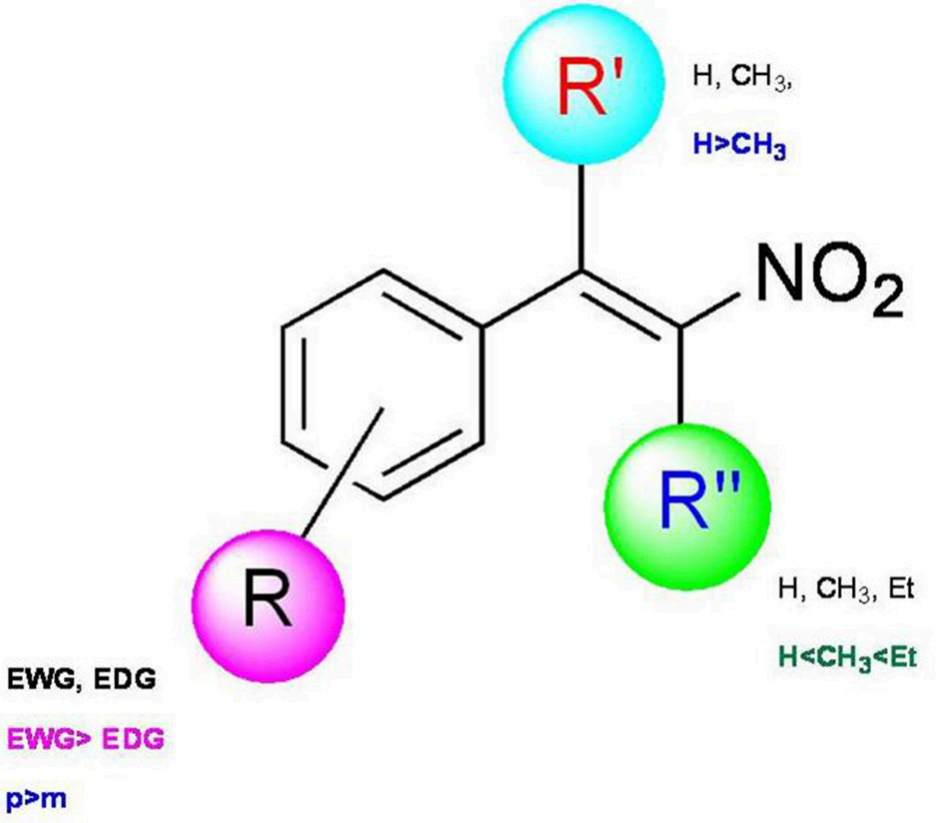
**Structure-activity relationship of β-nitrostyrenes**.

In this regard we have selected antimicrobial β-nitrostyrenes to evaluate their anti-leishmanial potentials. Inspired by the naturally occurring β-nitrostyrenes, a focused library of thirty β-nitrostyrenes was synthesized by varying nature and position of substitution over the aromatic ring and the nature of alkyl chain at β-position. All the synthesized compounds were tested for their antibacterial, antifungal, and antileishmanial activities. Close congeners (compound 9, 12, 14, and 18) of the naturally occurring β-nitrostyrenes were found to be most active amongst the compounds tested (Figure 7) against both the both forms of *L. donovani* i.e., Promastigotes and amastigotes. These compounds have demonstrated more or less similar degree of activity against the both forms (Promastigotes and amastigotes). From the results a SAR can be clearly depicted based on the substitution of aromatic moiety and the nature of the alkyl side chain on α and β positions of the β-nitrostyrenes (Figure 8). From the data it is clearly understood that the alkyl substitution at β position highly influences the biological activity against *L. donovani* promastigotes and amastigotes. Increase in the chain length leads to the improvement in the activity. Ethyl substitution at β position shows the highest degree of anti-leishmanial activity. The order of activity with respect to alkyl substitution at β position is H < Me < Et.

The activity has been also altered moderately based on the nature and position of the substituent on the aromatic ring. *Para*-substituted compounds were found to be more active than *meta*-substituted compounds. Nitro substitution on aromatic ring exerted better activity than the electron donor groups. Substitution of nitro group at para/meta position with ethyl group at β position highly enhanced the activity (compounds 9 and 12, IC_50_ = 23.40 ± 0.71 and 37.83 ± 3.74 nM against promastigotes and 30.5 ± 3.42, 21.46 ± 0.96 nM against amastigotes respectively). Among electron donor substituents 4-OMe, 4-OH substituents exhibited better activity. Compound 14 with methoxy substitution at the *para*-position of the aromatic system and methyl group at β-position of the β-nitrostyrene have shown the prominent anti-leishmanial activity with IC_50_ 40.50 ± 1.47 and 26.43 ± 2.71 nM (against promastigotes and amastigotes respectively) whereas ethyl substitution at β-position reduced the activity (compound 15). Introduction of another methoxy group at the ortho position of 4-methoxy β-methyl β-nitrostyrenes (23) decreased the activity (showed moderate activity) while 3,4-dimethoxy β-nitrostyrene (22) and 3,4-dimethoxy β-ethyl β-nitrostyrenes (24) were found to be inactive.

Hydroxy group at *para*-position of the aromatic ring with ethyl substitution at β-position of β-nitrostyrene (Compound 18) resulted in the significant anti-leishmanial activity with IC_50_ 55.66 ± 2.84 nM when compared to *meta*-substitution (compound 21) (IC_50_ 28.42 ± 3.11 μM) against promastigates. Compound 18 also exhibited the similar activity profile against amastigotes with IC_50_ 61.63 ± 8.02 nM. While 4-hydroxy and 3-hydroxy β-nitrostyrenes with methyl substitution at β-position (compounds 17 and 20) were moderately active (IC_50*s*_ 39.36 ± 1.36 and 63.24 ± 2.06 μM respectively). Methyl substitution at α-position of the 4-hydroxy β-methyl /3-hydroxy β-ethyl nitrostyrenes (compounds 25, 26) resulted in the loss of activity. Other β-ethyl nitrostyrenes with Cl, N, N-dimethyl amino and 2-hydroxy-3-ethoxy substitutions (1–6, 27) were found to be inactive. Hetero aromatic nitro vinyl derivatives (indole based) were also been evaluated for their anti-leishmanial potentials. Irrespective of the β-chain elongation these compounds (28–30) were found to be inactive. Toxicity studies against macrophages reveal that these compounds were found to be insignificantly toxic when compared to standard AmB even at 25 μM concentrations.

NO estimation experiments further reveals the moderate increase in the NO expression levels. Among the compounds tested, compounds 9 and 12 moderately increased the NO levels while NO levels were mildly induced by compound 14.

## Conclusion

In conclusion, a focused library of thirty β-nitrostyrenes has been synthesized and was tested against eight reference strains of selected pathogenic organisms and *L. donovani* promastigotes. Some of these compounds showed a broad spectrum of antimicrobial activity. Compounds 9, 12, 14, and 18 were found to be highly active against both forms of *L. donovani* promastigotes and intracellular amastigotes (nM range) and comparable with the standard drug AmB. Further these compounds were found to be insignificantly toxic against mammalian macrophages even at a concentration of 25 μM. With the credentials of high degree of activity, safety profiles, and economic methods for the synthesis, this class of compounds may be potential candidates for further drug development against leishmaniasis. Detailed investigation of mechanistic studies is in progress.

## Author contributions

SS: Design and synthesis of the compounds and wrote the paper. MI: Designed anti-leishmanial experiments and analyzed the data. GC: Designed anti-leishmanial experiments and analyzed the data. IA: Designed anti-microbial experiments (anti-bacterial and fungal) and analyzed the data. FN: Synthesis of the compounds. MZ: Analysis of compounds. FA: Designed anti-leishmanial experiments and analyzed the data.

### Conflict of interest statement

The authors declare that the research was conducted in the absence of any commercial or financial relationships that could be construed as a potential conflict of interest.
