# Supplementary material for: β-Nitrostyrenes as Potential Anti-leishmanial Agents
Source: Front Microbiol. 2016 Sep 1;7:1379. doi: 10.3389/fmicb.2016.01379 (PMC5007854; doi:10.3389/fmicb.2016.01379)
Supplement: Supplementary file 1 [file DataSheet1.doc]

**β–nitrostyrenes as potential anti–leishmanial agents**

Syed Shafi^1*^, Mohammad Islamuddin^2,3^, Garima Chouhan^2^, Intzar Ali^4^, Faatima Naaz^5^, K. C. Sharma^5^, M. S. Zaman^5^, Farhat Afrin^6,2*^

^1^Department of Chemistry, Faculty of Science, Hamdard University, New Delhi–110 062, India

^2^Parasite Immunology Lab., Department of Biotechnology, Faculty of Science, Hamdard University, New Delhi–110 062, India

^3^Molecular Virology and Vaccinology Lab., Department of Biotechnology, Faculty of Science, Hamdard University, New Delhi–110 062, India

^4^Membrane Biology Laboratory, School of Life Sciences, Jawaharlal Nehru University, New Delhi–110 067, India

^5^Department of Pharmaceutical Chemistry, F/o Pharmacy, Jamia Hamdard, New Delhi-62.

^6^Department of Medical Laboratories Technology, Faculty of Applied Medical Sciences, Taibah University, Medina–30 001, Saudi Arabia.

**^*^Corresponding authors.**

*E–mail addresses*:

syedshafi@jamiahamdard.ac.in (Syed Shafi),

[afrin_farhat@yahoo.co.in](mailto:afrin_farhat@yahoo.co.in) (Farhat Afrin),

rp47@mail.jnu.ac.in (Rajendra Prasad).

**Contents**

^1^H, ^13^C NMR and Mass spectra of compound **3, 12** and **29** ….………....…2–7

**^1^H- NMR of compound-12**


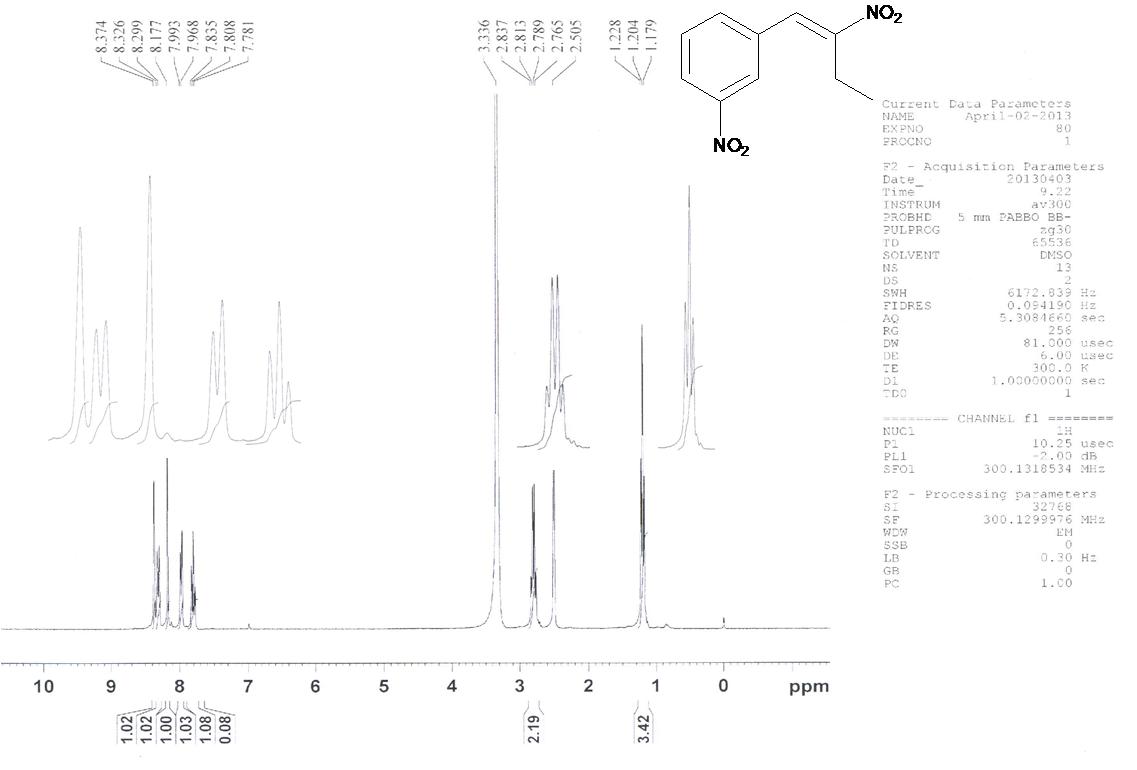


**^13^C- NMR of compound-12**


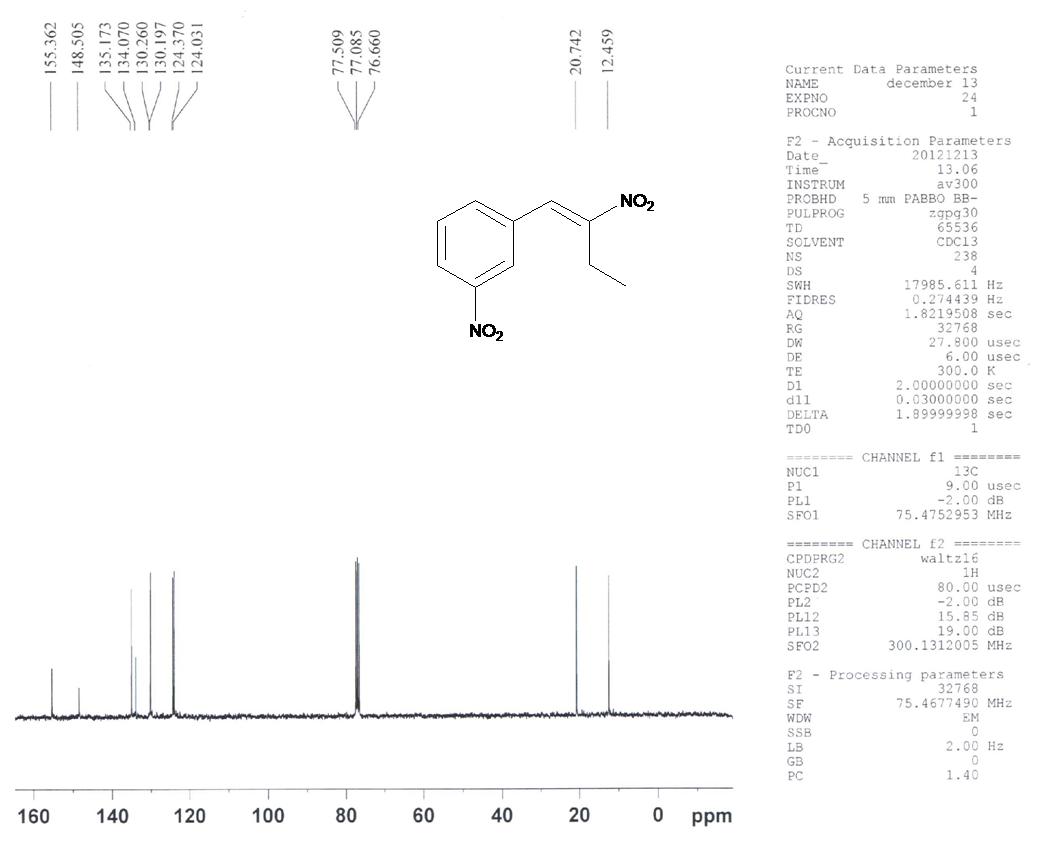


**ESI-MS of Compound-12**


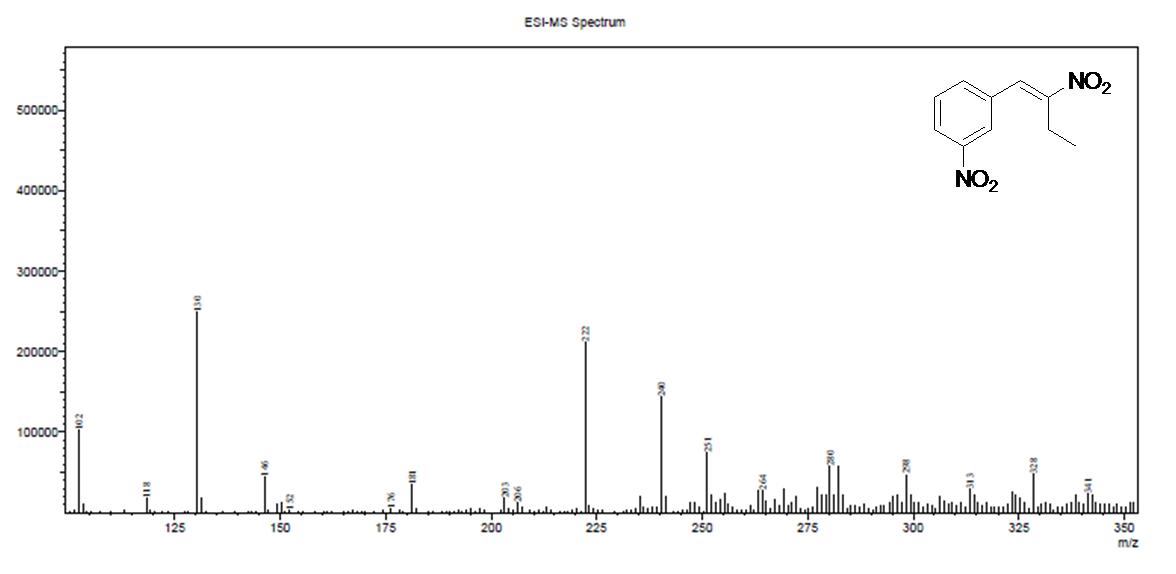


**^1^H- NMR of compound-3**


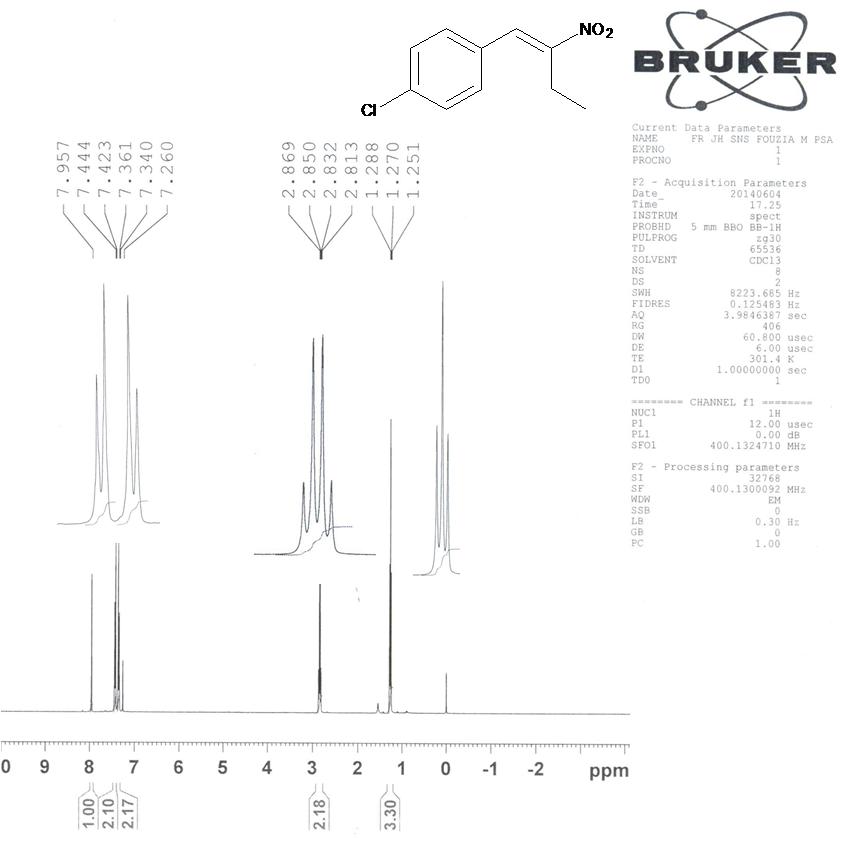


**^13^C- NMR of compound-3**


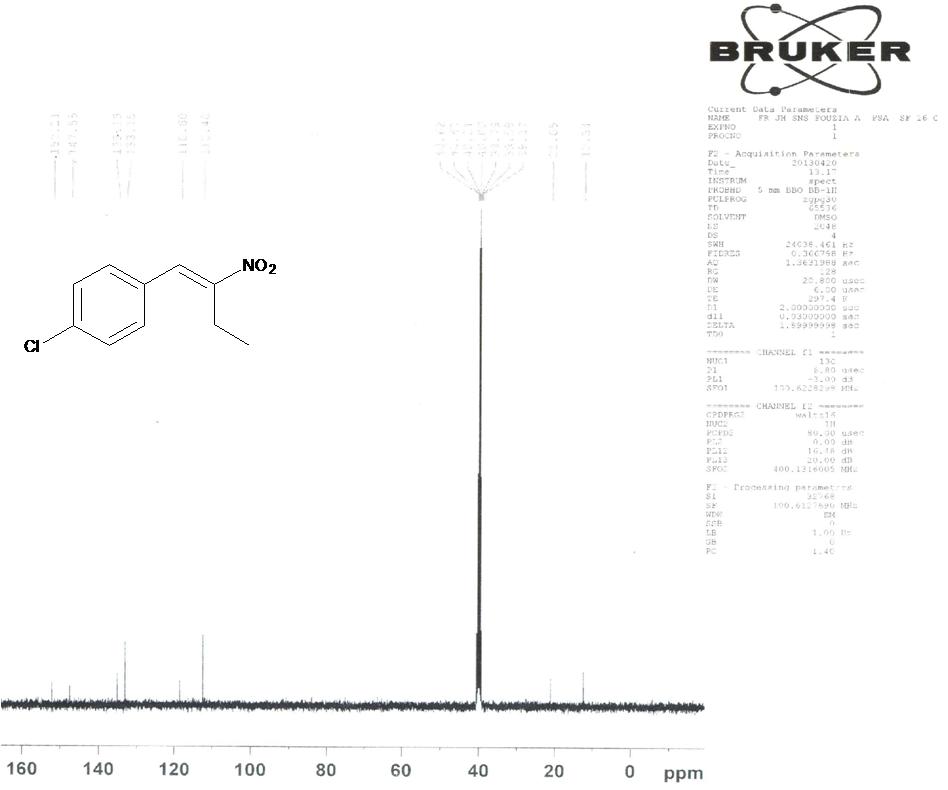


**^1^H- NMR of compound-29**


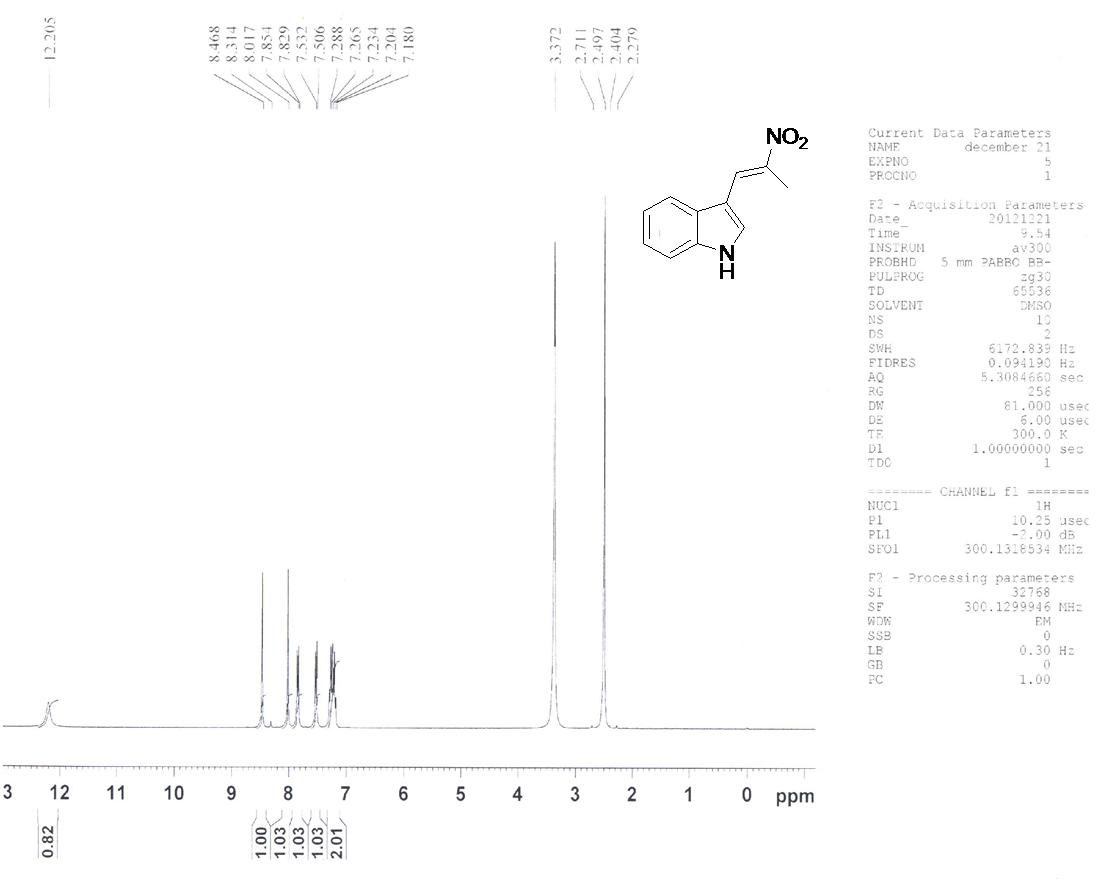


**^13^C- NMR of compound-29**


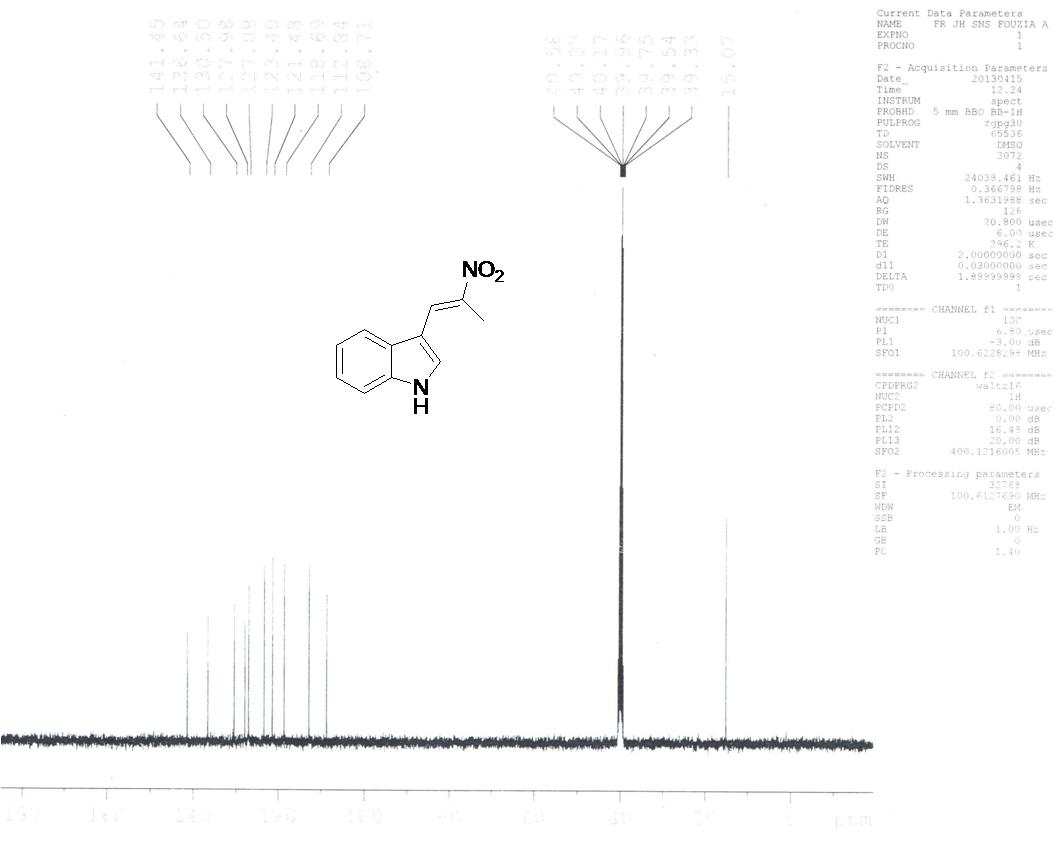


**ESI-MS of Compound-29**


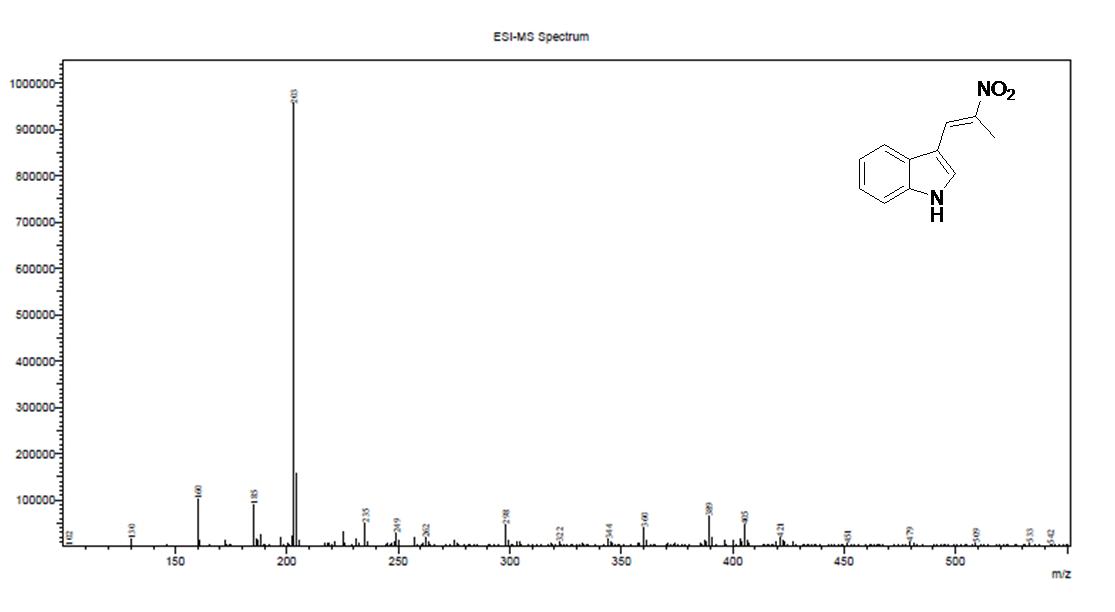


Syed Shafi1*, Mohammad Islamuddin2, 3, Garima Chouhan2, Intzar Ali 4, Faatima Naaz 5,

Kalicharan Sharma5, Mohammad S. Zaman5 and Farhat Afrin2, 6*

1Medicinal Chemistry Lab, Department of Chemistry, Faculty of Science, Hamdard University, New Delhi, India, 2 Parasite

Immunology Lab, Department of Biotechnology, Faculty of Science, Hamdard University, New Delhi, India, 3Molecular

Virology and Vaccinology Lab, Department of Biotechnology, Faculty of Science, Hamdard University, New Delhi, India,

4Membrane Biology Laboratory,School of Life Sciences, Jawaharlal Nehru University, New Delhi, India, 5Medicinal Chemistry

Lab, Department of Pharmaceutical Chemistry, Faculty of Pharmacy, Jamia Hamdard, New Delhi, India, 6 Department of

Medical Laboratories Technology, Faculty of Applied Medical Sciences, Taibah University, Medina, Saudi Arabia
